# Thiolactones and Δ^8,9^-Pregnene Steroids from the Marine-Derived Fungus *Meira* sp. 1210CH-42 and Their *α*-Glucosidase Inhibitory Activity

**DOI:** 10.3390/md21040246

**Published:** 2023-04-16

**Authors:** Hee Jae Shin, Min Ah Lee, Hwa-Sun Lee, Chang-Su Heo

**Affiliations:** 1 Marine Natural Products Chemistry Laboratory, Korea Institute of Ocean Science and Technology, 385 Haeyang-ro, Yeongdo-gu, Busan 49111, Republic of Korea; minah@kiost.ac.kr (M.A.L.); hwasunlee@kiost.ac.kr (H.-S.L.); science30@kiost.ac.kr (C.-S.H.); 2 Department of Marine Biotechnology, University of Science and Technology (UST), 217 Gajungro, Yuseong-gu, Daejeon 34113, Republic of Korea; 3 Department of Chemistry, Pukyong National University, Busan 48513, Republic of Korea

**Keywords:** marine fungus, natural product, *Meira* sp., thiolactone, pregnene steroid, epimer, stereochemistry, *α*-glucosidase inhibitor

## Abstract

The fungal genus *Meira* was first reported in 2003 and has mostly been found on land. This is the first report of second metabolites from the marine-derived yeast-like fungus *Meira* sp. One new thiolactone (**1**), along with one revised thiolactone (**2**), two new Δ^8,9^-steroids (**4**, **5**), and one known Δ^8,9^-steroid (**3**), were isolated from the *Meira* sp. 1210CH-42. Their structures were elucidated based on the comprehensive spectroscopic data analysis of 1D, 2D NMR, HR-ESIMS, ECD calculations, and the pyridine-induced deshielding effect. The structure of **5** was confirmed by oxidation of **4** to semisynthetic **5**. In the *α*-glucosidase inhibition assay, compounds **2**–**4** showed potent in vitro inhibitory activity with IC_50_ values of 148.4, 279.7, and 86.0 μM, respectively. Compounds **2**–**4** exhibited superior activity as compared to acarbose (IC_50_ = 418.9 μM).

## 1. Introduction

Fungi constitute one of the largest groups of organisms. Fungal-derived natural products (NPs) are pharmaceutically abundant, with several important biological applications ranging from highly potent toxins to approved drugs [1]. In particular, secondary metabolites obtained from marine fungi have garnered significant interest due to their unique chemical structures and potential biomedical applications [1,2]. While the number of cultivable marine fungi is extremely low (1% or less) compared to their global biodiversity [1,3], more than 1000 molecules have been reported and characterized from marine fungi, including alkaloids, lipids, peptides, polyketides, prenylated polyketides, and terpenoids [4,5,6,7]. Most research on secondary metabolites produced by marine fungi has primarily focused on a few genera, including *Penicillium*, *Aspergillus*, *Fusarium*, and *Cladosporium* [8,9]. Research into natural products derived from marine fungi is continually expanding, and as a result, a broader range of genera is now being investigated, with a particular focus on those associated with unique substrates and previously unexplored habitats [10,11,12].

In 2003, the genus *Meira* was first reported, namely *M. geulakonigii* and *M. argovae,* as a novel basidiomycetous [13]. *M. geulakonigii* was isolated from the citrus rust mite on pummelo (*Citrus grandis*), and *M. argovae* originated from a carmine spider mite on the leaves of castor bean (*Ricinus communis*) [8]. These *Meira* species have a similar morphology to yeast-like fungi. Nonetheless, the phylogenetic analysis of rDNA sequence data has identified *Meira* as a member of the Brachybasidiaceae family within the Exobasidiales, which is classified under the Ustilaginomycetes (Basidiomycota) in the Exobasidiomycetidae group [14]. *M. geulakonigii* has been used successfully as a biological control agent against citrus and other phytophagous mites, as well as powdery mildew fungi [13,15,16,17]. A potential biocontrol agent against five mite species has been demonstrated for *M. argovae* [18]. Recently, *M. nicotianae* came from the rhizosphere of tobacco root, and that strain has the capability to promote plant growth possible in similar ways as plant growth-promoting fungi and arbuscular mycorrhizal fungi [19].

**Figure 1:**
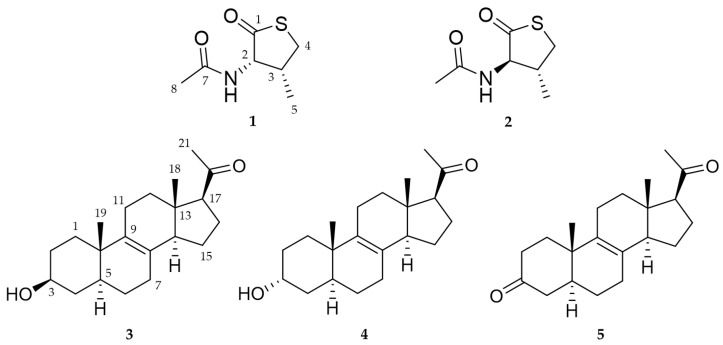
Structures of **1**–**5** from the marine fungus strain *Meira* sp. 1210CH-42.

In this study, we isolated a yeast-like fungal species from a seawater sample. Phylogenetic analysis of ITS rDNA indicated that strain 1210CH-42 is closely related to other *Meira* species: *Meira* sp. M40, *M. nashicola* CY-1, and *M. miltonrushii* NIOSN-SK46-S121. So far, there are only a few reports on the isolation of *Meira* strains, but natural products from the genus *Meira* have not been investigated. This is the first report on the secondary metabolites from the marine-derived yeast-like fungus *Meira*. Herein, we report the isolation, structure elucidation, *α*-glucosidase inhibitory activity of **1**–**5**, and the structure revision of **2** isolated from the *Meira* strain 1210CH-42 (Figure 1).

## 2. Results and Discussion

### 2.1. Structure Elucidation of New Compounds

**Figure 2:**
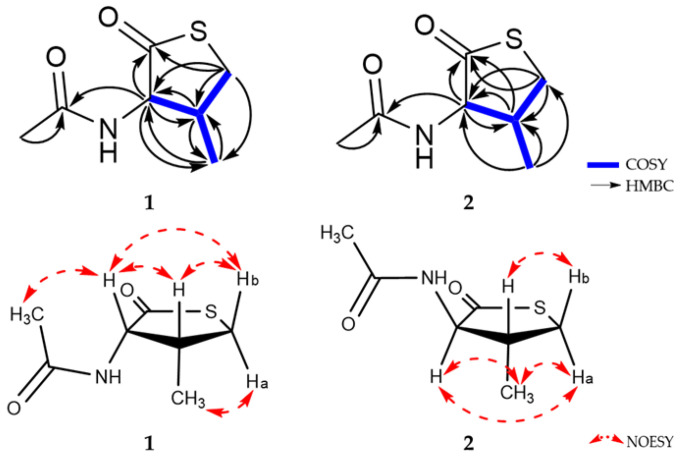
^1^H-^1^H COSY and key 2D NMR correlations of **1** and **2**.

Compound **1** was obtained as a white amorphous powder, and its molecular formula was determined to be C_7_H_11_NO_2_S by HR-ESIMS, with three degrees of unsaturation. The ^1^H and ^13^C NMR data of **1** are summarized in Table 1. The ^1^H NMR spectrum of **1** in CD_3_OD revealed two methine protons (*δ*_H_ 4.79 and 2.86), one methylene proton (*δ*_H_ 3.64 and 3.10), and two methyl protons (*δ*_H_ 2.03 and 1.04). The ^13^C NMR and HSQC spectra showed the presence of seven signals, including two carbonyl carbons (*δ*_C_ 206.5 and 173.8), two methines (*δ*_C_ 63.9 and 36.0), one methylene (*δ*_C_ 35.9) and two methyl carbons (*δ*_C_ 22.4 and 13.0). The planar structure of **1** was elucidated by analysis of ^1^H-^1^H COSY and HMBC correlations (Figure 2). The COSY correlations from H-2 (*δ*_H_ 4.79)/H-3 (*δ*_H_ 2.86), H-3 (*δ*_H_ 2.86)/H-4 (*δ*_H_ 3.64), and H-3 (*δ*_H_ 2.86)/H-5 (*δ*_H_ 1.04) were observed. In addition, the HMBC correlations from H-2 (*δ*_H_ 4.79) to C-1 (*δ*_C_ 206.5)/C-3 (*δ*_C_ 36.0)/C-5 (*δ*_C_ 13.0)/C-7 (*δ*_C_ 173.8), H-4 (*δ*_H_ 3.10 and 3.64) to C-1 (*δ*_C_ 206.5)/C-2 (*δ*_C_ 63.9)/C-5 (*δ*_C_ 13.0) and H-8 (*δ*_H_ 2.03) to C-7 (*δ*_C_ 206.5) suggested that **1** has a ring system, and confirmed the planar structure of **1**.

**Figure 3:**
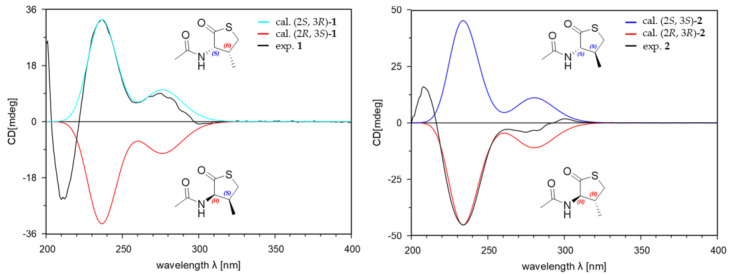
Experimental CD spectra and the calculated ECD spectra of **1** and **2**.

Detailed analysis of ^3^*J*_H,H_ coupling constants and 1D NOESY data determined the relative configuration of **1**. The relative stereochemistry of C-2 could be established by the observation of strong selective 1D NOESY correlations between H-2 and H-3/H-4b, between H-4b and H-2/H-3, and between H-5 and H-4a (Figure 2). These correlations suggested that the relative configurations of C-2 and C-3 must be *cis* rather than *trans*-configuration in **1**. Thus, the relative configuration of **1** could be assigned as 2S*, 3R*. To determine the absolute configuration of **1**, the theoretical electronic circular dichroism (ECD) spectra of **1** and its enantiomer were calculated. The experimental ECD spectrum of **1** showed a good agreement with the calculated ECD spectrum of the 2*S*, 3*R*-isomer (Figure 3). Therefore, the structure of **1** was elucidated to be a 2*S*-acetamide-3*R*-methyl-thiolactone.

Compound **2** was isolated as a white amorphous powder. The molecular formula of **2** was the same as that of **1** (C_7_H_11_NO_2_S) based on the HR-ESIMS data. Furthermore, the 1D NMR data of **2** were also similar but not identical to those of **1** (Table 1). The planar structure of **2** was determined to be the same as **1** by analysis of ^1^H-^1^H COSY and HMBC data (Figure 2). However, the ^1^H and ^13^C chemical shifts of **2** were different from **1**, especially those for the chiral centers, suggesting that the stereochemistry of **2** might be different from **1**. The relative configuration of **2** was also determined by analysis of ^3^*J*_H,H_ coupling constants and selective 1D NOESY data. The relative stereochemistry of C-2 could be established through the observation of strong NOESY contacts between H-2 and H-4a/H-5, between H-4a and H-2/H-5, and between H-4b and H-3. A relatively large coupling constant was observed between H-2 and H-3 (^3^*J*_H,H_ = 12.5 Hz). Thus, the relative configurations of H-2 and H-3 had a *trans*-configuration in **2** (Figure 2). The *J*-based configurational analysis and NOESY measurements could not discriminate the possible relative configurations for (2S*, 3S*) or (2R*, 3R*). To solve this issue and to determine the absolute configuration of **2**, the ECD spectra of **2** and its enantiomer were calculated. The experimental ECD spectrum of **2** showed a good agreement with the calculated ECD spectrum of the 2*R*, 3*R*-isomer (Figure 3). Therefore, the structure of **2** was elucidated as an epimer of **1** and to be a 2*R*-acetamide-3*R*-methyl-thiolactone.

**Figure 4:**
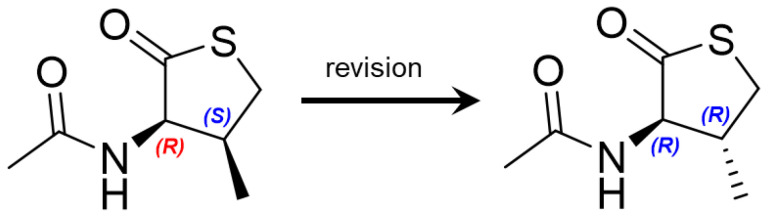
Reported and revised structures of **2**.

Notably, the ^1^H and ^13^C NMR data in CDCl_3_ of **2** were almost the same as those of the previously reported thiolactone with 2*R*, 3*S*-configuration isolated from a *Penicillium chrysogenum* (Table S1 and Figure S15) [20]. The reported compound with 2*R*, 3*S*-configuration possesses the same planar structure as **2** in this study. In the original paper for the compound with 2*R*, 3*S*-configuration, by the NOE correlation between H-3 (*δ*_H_ 2.24) and H-2 (*δ*_H_ 4.45), the authors insisted that the two protons were oriented on the same side of the ring system. However, its 1D NOE spectrum for the reported compound showed signals from H-3 (*δ*_H_ 2.24) to H-2/H-4/H-5/H-6 and NH, making it unclear to determine the orientation of H-3 to the same side of H-2 or not (Figures S15 and S16). Moreover, if the reported configuration is correct, H-2 and H-3 are in *syn* relation, and they should have a small scalar coupling constant, but H-2 in the reported thiolactone had a large coupling constant (12.5 Hz) as in the revised structure (Table S1). In this study, we carefully compared and checked the selective 1D NOESY data of **2** with those for the reported compound. As noted above, **2** exhibited strong NOE correlations from H-2 to H-5/ H-4a and from H-4b to H-3 but not from H-4b to H-2, suggesting that H-2 and H-5 are on the same face. Furthermore, the reported compound with 2*R*, 3*S*-configuration and **1** (2*S*, 3*R*-configuration) are enantiomers and should have the same but opposite-in-sign specific rotation values. However, the optical rotation values of the reported thiolactone and **1** were [α]${}_{D}^{25}$ +1.5 (*c* 0.1, MeOH) and [α]${}_{D}^{25}$ +60.0 (*c* 0.1, MeOH), respectively. Considering all these results, the structure of the reported compound (2*R*, 3*S*-configuration) should be revised to 2*R*-acetamide-3*R*-methyl-thiolactone (Figure 4).

Compound **3** was isolated as a white amorphous powder, and its molecular formula was determined to be C_21_H_32_O_2_. By the comparison of the ^1^H and ^13^C NMR (Table 2), HR-ESIMS, and optical rotation data of **3** with those reported previously in the literature, **3** was identified as a known compound, (+)-03219A, Δ^8,9^-3*β*-hydroxy-5*α*-17-acetyl steroid [21,22,23].

**Figure 5:**
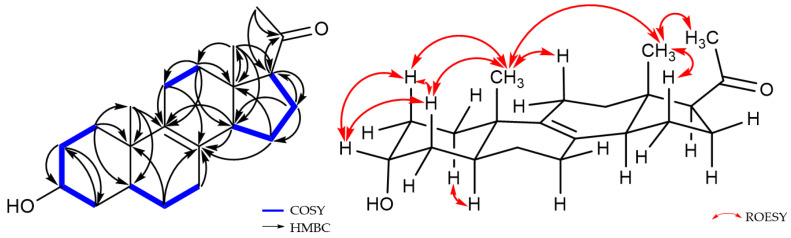
^1^H-^1^H COSY and key 2D NMR correlations of **4**.

**Figure 6:**
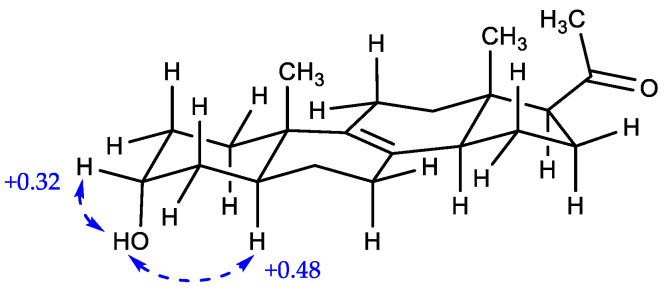
Pyridine-induced deshielding effects of **4** (*Δδ* = *δ*_H_ in C_5_D_5_N-*δ*_H_ in CD_3_OD).

Compound **4** was purified as a white amorphous powder, and its molecular formula was determined to be C_21_H_32_O_2_ by HR-ESIMS, which is identical to that of **3**, with 6 degrees of unsaturation. The ^1^H and ^13^C NMR data of **4** are summarized in Table 2. The ^1^H NMR data for **4** revealed the signals of three methyl groups (*δ*_H_ 0.57, 0.94, and 2.13), one oxymethine (*δ*_H_ 3.97), nine methylenes, and three *sp*^3^ methines. The ^13^C NMR and HSQC data of **4** exhibited 21 carbon signals containing three methyls (*δ*_C_ 13.2, 17.3, and 31.7), one oxymethine (*δ*_C_ 67.2), nine methylenes, two olefinic quaternary carbons (*δ*_C_ 129.0 and 137.2), two *sp*^3^ quarternary carbons (*δ*_C_ 37.6 and 45.1), and one ketone carbonyl carbon (*δ*_C_ 212.5). The planar structure of **4** was elucidated by ^1^H-^1^H COSY and HMBC data (Figure 5). The ^1^H-^1^H COSY correlations suggested the presence of four ^1^H-^1^H spin systems: from H-1 to H-4, from H-5 to H-7, from H-11 to H-12, and from H-14 to H-17. The HMBC correlations from H-6/H-7/H-11/H-14/H-15 to C-8 (*δ*_C_ 129.0) and from H-11/H-12/H-14/H-19 to C-9 (*δ*_C_ 137.2) indicated a double bond was located at C-8 and C-9. Additionally, the HMBC correlations from H-21 to C-17 (*δ*_C_ 63.5)/C-20 (*δ*_C_ 212.5) supported the assignment of an acetyl moiety connected to C-17 of the five-membered ring. The planar structure of **4** was the same as that of **3**, (+)-03219A [23], except for the difference in the chemical shifts around the oxymethine (*δ*_H_ 3.97 and *δ*_C_ 67.2) at C-3, suggesting that the stereochemistry of C-3 might be different from **3** (Figure 1 and Table 2). The stereochemistry of **4** was determined by analysis of the ROESY spectrum, 1D NOESY data, coupling constants, and the pyridine-induced deshielding effect. The relative configuration of **4** was confirmed by the ROESY correlations from H-3 to H-2a/H-2b/H-4, from H-19 to H-2b/H-4/H-11/H-18, and from H-18 to H-15/H-21 (Figure 5). The selective 1D NOE correlations were observed from H-3 to H-2a/H-2b/H-4/H-19 (Figure S27). Furthermore, the small coupling constant of H-3 at *δ*_H_ 3.97 (t, *J* = 2.8) was indicative of the C-3 hydroxyl group being axial from an examination of the Dreiding model (Table 2 and Figure 5) [24]. The significant deshielded chemical shifts of H_eq_-3 (*Δδ*_H_ = +0.32) and H_ax_-5 (*Δδ*_H_ = +0.48) in pyridine-*d*_5_ compared with those in CD_3_OD indicated that OH-3 and H-5 adopted *α*-orientation, supporting the identified orientation (Figure 6 and Figure S29) [25,26,27,28]. Consequently, the structure of **4** was determined as a new epimer of **3**, Δ^8,9^-3*α*-hydroxy-5*α*-17-acetyl steroid.

**Figure 7:**
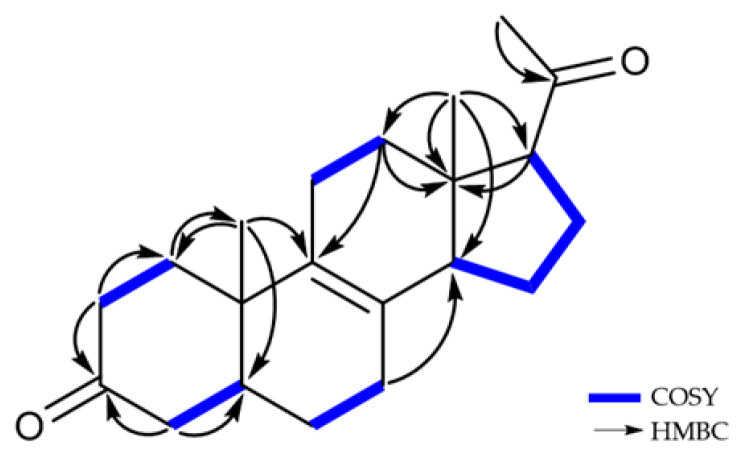
^1^H-^1^H COSY and key HMBC correlations of **5**.

Compound **5** was obtained as a white amorphous powder. The NMR data of **5** were similar to those of **4**, except for the absence of signals for the oxymethine at C-3 (*δ*_H_ 3.97 and *δ*_C_ 67.2) in **4** and the appearance of a ketone signal at C-3 (*δ*_C_ 214.6) in **5** (Table 2), revealing that **5** would be an oxidized form of **4**. The ^1^H and ^13^C NMR spectra, compared to those of **3** and **4**, showed the significantly deshielded chemical shifts of C-2 (*δ*_H_ 2.31/2.53 and *δ*_C_ 39.1) and C-4 (*δ*_H_ 2.11/2.40 and *δ*_C_ 45.7). Additionally, the HMBC correlations between H-2b (*δ*_H_ 2.53)/H-4 (*δ*_H_ 2.11/2.40) and C-3 (*δ*_C_ 214.6) determined the position of the ketone at C-3 (Figure 7). To clearly confirm the structure of **5**, **4** was oxidized to obtain the semisynthetic **5**. Both **5** and semisynthetic **5** exhibited identical ^1^H NMR, HSQC, and HMBC spectra (Figures S35, S36 and S37). The molecular formula of semisynthetic **5** was determined to be C_21_H_30_O_2_ by HR-ESIMS (*m*/*z* 337.2134 [M + Na]^+^, calcd. for C_21_H_30_O_2_Na, 337.2138). Based on these results, the structure of **5** was determined as a 3-keto derivative of **4**, with 7 degrees of unsaturation. Therefore, the structures of **5** and semisynthetic **5** were designated as Δ^8,9^-5*α*-3,20-dione-17-acetyl steroids.

### 2.2. α-Glucosidase Inhibitory Activities of Compounds

Compounds **1**–**4** were evaluated for *α*-glucosidase inhibitory activities (Table 3). Compound **4** exhibited the most significant inhibitory effect with an IC_50_ value of 86.0 μM, while **2** and **3** showed moderate activities with IC_50_ values of 148.4 and 279.7 μM, respectively. Further, **1** exhibited weak inhibitory activity at a concentration of 400 μM. The change in the stereochemistry of the compounds remarkably altered the *α*-glucosidase inhibitory activities. Compounds **1** and **2**, as well as **3** and **4**, are stereoisomers of each other. Compounds **2** and **4** showed stronger *α*-glucosidase inhibitory effects than **1** and **3**. It could be noted herein that the stereochemistry was important for *α*-glucosidase inhibitory activity.

## 3. Materials and Methods

### 3.1. General Experimental Procedures and Reagents

NMR spectra were acquired with a Bruker AVANCE III 600 spectrometer (Bruker BioSpin GmbH, Rheinstetten, Germany) with a 3 mm probe operating at 600 MHz (^1^H) and 150 MHz (^13^C). Chemical shifts were expressed in ppm with reference to the solvent peaks (*δ*_H_ 3.31 and *δ*_C_ 49.15 ppm for CD_3_OD, *δ*_H_ 7.26 and *δ*_C_ 77.26 ppm for CDCl_3_). UV spectra were recorded with a Shimadzu UV-1650PC spectrophotometer (Shimadzu Corporation, Kyoto, Japan). IR spectra were obtained on a JASCO FT/IR-4100 spectrophotometer (JASCO Corporation, Tokyo, Japan). Optical rotations were measured with a Rudolph analytical Autopol III S2 polarimeter (Rudolph Research Analytical, Hackettstown, NJ, USA). LR-ESIMS data were obtained with an ISQ EM mass spectrometer (Thermo Fisher Scientific Korea Ltd., Seoul, Republic of Korea). HR-ESIMS data were obtained with a Waters SYNPT G2 Q-TOF mass spectrometer (Waters Corporation, Milford, CT, USA) at Korea Basic Science Institute (KBSI) in Cheongju, Republic of Korea and a Sciex X500R Q-TOF spectrometer (Framingham, MA, USA). ECD spectra were recorded with a JASCO J-1500 polarimeter at the Center for Research Facilities, Changwon National University, Changwon, Republic of Korea. HPLC was performed using a BLS-Class pump (Teledyne SSI, Inc., State College, PA 16803, USA) with Shodex RI-201H refractive index detector (Shoko Scientific Co., Ltd., Yokohama, Japan). Columns for HPLC were YMC-ODS-A (250 mm × 10 mm, 5 μm; and 250 mm × 10 mm, 5 μm) and YMC-Triart (250 mm × 10 mm, 5 μm; and 250 mm × 10 mm 5 μm). C_18_-reversed-phase silica gel (YMC-Gel ODS-A, 12 nm, S-75 μm) was used for open-column chromatography. Organic solvents were purchased as HPLC grade, and ultrapure waters were obtained from the Milipore Mili-Q Direct 8 system (Milipore S.A.S. Molsheim, France). The reagents used in the bioassay were purchased from Sigma-Aldrich (Merck Korea, Seoul, Republic of Korea) and Tokyo Chemical Industry (TCI Co., Ltd., Tokyo, Japan).

### 3.2. Fungal Strain and Fermentation

The strain 1210CH-42 was isolated from a seawater sample collected at Chuuk Islands, Federated States of Micronesia, in 2010. The seawater sample was filtered, concentrated, and diluted (10^−1^ and 10^−2^) with sterile seawater under aseptic conditions. Then the diluted sample was spread on Bennett’s agar plates (1% D-glucose, 0.2% tryptone, 0.1% yeast extract, 0.1% beef extract, 0.5% glycerol, 1.7% agar, sea salt 32 g/L, pH 7.0). The plates were incubated for 7 days at 28 °C, and the single colony of the strain 1210CH-42 was collected. The fungus was identified as *Meira* sp. (GenBank accession number OQ693946) by DNA amplification and sequencing of the ITS region of the rRNA gene. The used primers were ITS4 (TCCTCCGCTTATTGATATGC) and ITS5 (GGAAGTAAAAGTCGTAACAAG G). The cultures of the strain 1210CH-42 were performed in modified Bennett’s broth medium (1% D-glucose, 0.2% tryptone, 0.1% yeast extract, 0.1% beef extract, 0.5% glycerol, sea salt 10 g/L, pH 7.0). A seed culture was prepared from a spore suspension of the strain 1210CH-42 by inoculating into 1 L flasks and incubating it at 28 °C for 5 days on a rotary shaker at 120 rpm. The seed culture was inoculated aseptically into 2 L flasks (total 32 flasks) containing 1.0 L of medium and a 20 L fermenter containing 18 L of sterilized culture medium (0.1% *v*/*v*), respectively. The large-scale fermentation was done under the same conditions as the seed culture for 8 days and then harvested.

### 3.3. Extraction and Isolation of Compounds ***1***–***5***

The culture broth (total 50 L) of the strain 1210CH-42 was harvested by high-speed centrifugation (60,000 rpm), and then the supernatant was extracted two times with ethyl acetate (100 L). The EtOAc extract was evaporated to afford a crude extract (3.05 g). The crude extract was subjected to ODS open column chromatography (YMC Gel ODS-A, 12 nm, S75 μm) followed by stepwise gradient elution with MeOH/H_2_O (*v*/*v*) (20:80, 40:60, 60:40, 80:20, and 100:0) as eluent. The 20% MeOH fraction was purified by a reversed-phase HPLC (YMC ODS-A column, 250 × 10 mm i.d., 5 μm; 10% MeOH in H_2_O; flow rate: 1.5 mL/min; detector: RI) to yield **1** (2.9 mg, *t*_R_ 44.0 min). Peak 10 from the 20% MeOH fraction was further purified by a reversed-phase HPLC (YMC ODS-A column, 250 × 10 mm i.d., 5 μm; 5% MeOH in H_2_O; flow rate: 1.5 mL/min; detector: RI) to yield **2** (0.6 mg, *t*_R_ 64.0 min). The 80% MeOH fraction was purified by a reversed-phase HPLC (YMC ODS-A column, 250 × 10 mm i.d., 5 μm; 70% MeOH in H_2_O; flow rate: 1.5 mL/min; detector: RI) to yield **3** (0.6 mg, *t*_R_ 84.0 min), **4** (2.1 mg, *t*_R_ 95.5 min), and **5** (0.3 mg, *t*_R_ 79.0 min).

Compound **1**: White amorphous powder; [*α*${]}_{D}^{25}$ +60.0 (*c* 0.1, MeOH); UV (MeOH) *λ*_max_ (log *ε*) 204 (3.64), 235 (3.33) nm; IR (MeOH) *ν*_max_ 3296, 2940, 1667, 1548, 1448, 1021 cm^−1^; ^1^H and ^13^C NMR data (CD_3_OD), see Table 1; HR-ESIMS *m*/*z* 196.0408 [M + Na]^+^, calcd. for C_7_H_11_NO_2_NaS, 196.0408.

Compound **2**: White amorphous powder; [*α*${]}_{D}^{25}$ +10.0 (*c* 0.1, MeOH); UV (MeOH) *λ*_max_ (log *ε*) 206 (3.88), 234 (3.69) nm; IR (MeOH) *ν*_max_ 3275, 2933, 1700, 1650, 1548, 1448, 1021 cm^−1^; ^1^H and ^13^C NMR data (CD_3_OD), see Table 1; HR-ESIMS *m*/*z* 196.0406 [M + Na]^+^, calcd. for C_7_H_11_NO_2_NaS, 196.0408.

Compound **3**: White crystalline needles; [*α*${]}_{D}^{25}$ +86.0 (*c* 0.1, MeOH); UV (MeOH) *λ*_max_ (log *ε*) 202 (4.10) nm; IR (MeOH) *ν*_max_ 3371, 2925, 2855, 1703, 1452, 1357, 1032 cm^−1^; ^1^H and ^13^C NMR data (CD_3_OD), see Table 2; HR-ESIMS *m*/*z* 339.2297 [M + Na]^+^, calcd. for C_21_H_32_O_2_Na, 339.2300.

Compound **4**: White crystalline needles; [*α*${]}_{D}^{25}$ +97.3 (*c* 0.1, MeOH); UV (MeOH) *λ*_max_ (log *ε*) 202 (3.96) nm; IR (MeOH) *ν*_max_ 3286, 2925, 2870, 1703, 1452, 1353, 1025 cm^−1^; ^1^H and ^13^C NMR data (CD_3_OD), see Table 2; HR-ESIMS *m*/*z* 339.2301 [M + Na]^+^, calcd. for C_21_H_32_O_2_Na, 339.2300.

Compound **5**: White amorphous; [*α*${]}_{D}^{25}$ +63.3 (*c* 0.1, MeOH); UV (MeOH) *λ*_max_ (log *ε*) 204 (3.86) nm; IR (MeOH) *ν*_max_ 3378, 2933, 2866, 1707, 1456, 1367, 1036 cm^−1^; ^1^H and ^13^C NMR data (CD_3_OD), see Table 2.

Oxidation of **4**. To a compound **4** (2.0 mg, 6.32 μmol) in anhydrous CH_2_Cl_2_ (0.5 mL) was added Dess-Martin reagent (8.04 mg, 18.96 μmol) at 0 °C. The mixture was stirred at r.t. for 24 h under N_2_ gas. The solution was washed with 5% NaHCO_3_ and brine and concentrated under reduced pressure [29,30]. Then the reactant was partitioned with EtOAc and H_2_O. The EtOAc layer was concentrated, and subjected to a reversed-phase HPLC (YMC-Triart C_18_ column, 250 × 10 mm i.d., 5 μm; 70% MeOH in H_2_O; flow rate: 2.0 mL/min; detector: RI) to yield semisynthetic **5** (0.5 mg): white amorphous solid; ^1^H NMR (600 MHz, CD_3_OD, representative signals) *δ*_H_ 2.71 (t, *J* = 8.7 Hz, 1H), 2.53–2.31 (m, 2H), 2.40–2.08 (t, *J* = 14.6, o. l, 2H), 2.30 (o. l, H), 2.23 (o. l, 2H), 2.14 (s, 3H), 2.21–1.71 (o. l, 2H), 2.07 (o. l, 2H), 1.81 (m, 2H), 1.70–1.47 (o. l, 2H), 1.56 (o. l, 2H), 1.45 (o. l, 2H), 1.18 (s, 3H), 0.60 (s, 3H); ^13^C NMR data from HMBC spectrum (CD_3_OD, representative signals) *δ*_C_ 214.5, 212.3, 135.6, 130.4, 63.5, 53.2, 45.7, 45.0, 44.4, 39.1, 38.1, 37.2, 31.7, 28.4, 26.7, 25.3, 24.3, 24.1, 23.7, 17.5, 13.1; HR-ESIMS *m*/*z* 327.2134 [M + Na]^+^, calcd. for C_21_H_30_O_2_Na, 327.2138.

### 3.4. Computational Analysis

The initial geometry optimization and conformational searches were generated using the Conflex 8 (Rev. B, Conflex Corp., Tokyo, Japan). The optimization and calculation for ECD were carried out using the Gaussian 16 program (rev. B.01, Gaussian Corp., Wallingford, C.T., USA). Conformational searches were performed using MMFF94s force field calculations with a 10 kcal/mol search limit. The conformers were optimized using the ground state method at the B3LYP/6-311+G (d, p) level in MeOH with a default model for ECD. The theoretical calculations of ECD spectra were performed using TD-SCF at the B3LYP /6-311+G (d, p) level in the gas phase. The ECD spectra were simulated by SpecDis (v. 1.71) using *σ* = 0.30–0.50 eV. All calculated curves were shifted to +10 nm to simulate experimental spectra better.

### 3.5. Measurement of α-Glucosidase Inhibitory Activity

The evaluation of *α*-glucosidase inhibitory activity was performed with reference to previously reported literature [31,32]. All the assays were carried out under 0.1 M PBS buffer (pH 7.4, Sigma). The samples (10 mM) were dissolved with DMSO (Sigma) and diluted into gradient concentrations with PBS buffer. The pre-reaction mixture consisted of the 130 μL sample with 30 μL *α*-glucosidase solution (0.2 U/mL, Sigma) and shaken well, then added to a 96-well plate and placed at 37 °C for 10 min in an incubator. Subsequently, 40 μL of 5 mM *p*-nitrophenyl-*α*-D-glucopyranoside (*p*NPG, TCI) was added and further incubated at 37 °C for 20 min. Finally, the *α*-glucosidase inhibitory activity was determined by measuring the release of *p*NPG at 405 nm of the microplate reader. The negative control was prepared by adding PBS buffer instead of the sample in the same way as the test. The blank was prepared by adding PBS buffer instead of *p*NPG using the same method. Acarbose was used as the positive control, and experiments were carried out in triplicate.

## 4. Conclusions

In summary, one new thiolactone (**1**), along with one revised thiolactone (**2**), two new Δ^8,9^-steroids (**4**, **5**), and one known Δ^8,9^-steroid (**3**), were isolated from the marine-derived fungus *Meira* sp. 1210CH-42. The absolute configurations of **1** and **2** were determined by analysis of the selective 1D NOESY and ECD data. Compounds **1** and **2** were identified as a pair of acetamide epimers at C-2. While compounds **3** and **4** were identified as epimers for the hydroxyl group at C-3, which was confirmed by analysis of ^1^H NMR, ROESY, 1D NOESY, coupling constants, and the pyridine-induced deshielding effect. In addition, the structure of **5** was obtained as the 3-keto derivative of **3**. Compounds **1**–**4** were screened for their *α*-glucosidase inhibitory activity preliminarily. Compound **4** exhibited intense activity with an IC_50_ value of 86.0 μM. Furthermore, compounds **2** (IC_50_ = 148.4 μM) and **3** (IC_50_ = 279.7 μM) demonstrated superior activity as compared to acarbose (IC_50_ = 418.9 μM). To the best of our knowledge, this is the first report of new bioactive metabolites with potent *α*-glucosidase inhibitory activity from the yeast-like fungus *Meira*. These results show that *Meira* sp. 1210CH-42 produces unique and diverse metabolites which have the potential for an anti-diabetic agent. The genus *Meira* is mostly found on land, and secondary metabolites from the marine-derived genus have not yet been reported. Therefore, further research is needed for the marine-derived fungus *Meira* sp. 1210CH-42 to discover novel secondary metabolites and investigate their biological properties.

## Figures and Tables

**Table 1:** ^1^H and ^13^C NMR data of **1** and **2** (600 MHz for ^1^H and 150 MHz for ^13^C, in CD_3_OD).

Position	1		2	
	*δ*_C_, Type	*δ*_H_, Mult. (*J* in Hz)	*δ*_C_, Type	*δ*_H_, Mult. (*J* in Hz)
1	206.5, C		206.5, C	
2	63.9, CH	4.79, d (6.6)	65.7, CH	4.29, d (12.5)
3	36.0, CH	2.86, m	40.2, CH	2.37, m
4a	35.9, CH_2_	3.10, dd (11.4, 2.2)	34.7, CH_2_	3.08, t (11.2)
4b		3.64, dd (11.4, 5.4)		3.34, d (11.2)
5	13.0, CH_3_	1.04, d (6.9)	17.5, CH_3_	1.20, d (6.5)
6-NH				
7	173.8, C		174.0, C	
8	22.4, CH_3_	2.03, s	22.6, CH_3_	2.02, s

**Table 2:** ^1^H and ^13^C NMR data of **3**–**5** (600 MHz for ^1^H and 150 MHz for ^13^C, in CD_3_OD).

Position	3		4		5	
	*δ*_C_, Type	*δ*_H_, Mult. (*J* in Hz)	*δ*_C_, Type	*δ*_H_, Mult. (*J* in Hz)	*δ*_C_, Type	*δ*_H_, Mult. (*J* in Hz)
1a	36.6, CH_2_	1.22, td (16.2, 5.2)	32.0, CH_2_	1.55, m	38.0, CH_2_	1.55, m
1b		1.80, o.l ^1^				
2a	32.4, CH_2_	1.42, o.l	37.1, CH_2_	1.48, o.l	39.1, CH_2_	2.31, o.l
2b		1.80, o.l		1.54, o.l		2.53, m
3	71.8, CH	3.53, m	67.2, CH	3.97, t (2.8)	214.6, C	
4a	39.2, CH_2_	1.31, o.l	30.0, CH_2_	1.68, m	45.7, CH_2_	2.11, o.l
4b		1.61, m				2.40, t (14.6)
5	42.3, CH	1.40, o.l	36.4, CH	1.86, m	44.4, CH	1.80, m
6a	26.8, CH_2_	1.39, o.l	26.7, CH_2_	1.32, m	26.7, CH_2_	1.47, o.l
6b		1.52, m		1.46, o.l		1.60, m
7	28.5, CH_2_	2.02, m	28.4, CH_2_	2.02, m	28.4, CH_2_	2.04, m
8	129.2, C		129.0, C		130.1, C	
9	136.3, C		137.2, C		135.6, C	
10	37.1, CH		37.6, CH		37.3, CH	
11a	24.0, CH_2_	2.15, m	23.6, CH_2_	2.13, o.l	24.1, CH_2_	2.20, m
11b		2.27, o.l		2.28, o.l		2.25, o.l
12a	37.3, CH_2_	1.69, m	37.3, CH_2_	1.70, o.l	37.2, CH_2_	1.70, o.l
12b		2.07, m		2.07, o.l		2.08, o.l
13	45.0, C		45.1, C		45.0, C	
14	53.3, CH	2.27, o.l	53.4, CH	2.30, o.l	53.2, CH	2.29, m
15a	25.3, CH_2_	1.42, o.l	25.3, CH_2_	1.42, o.l	25.3, CH_2_	1.45, o.l
15b		1.72, m		1.72, o.l		
16a	24.3, CH_2_	1.72, o.l	24.2, CH_2_	1.72, o.l	24.3, CH_2_	1.72, o.l
16b		2.21, m		2.21, m		2.21, m
17	63.5, CH	2.69, t (8.7)	63.5, CH	2.70, t (8.6)	63.5, CH	2.70, t (8.7)
18	13.2, CH_3_	0.57, s	13.2, CH_3_	0.57, s	13.2, CH_3_	0.60, s
19	18.3, CH_3_	0.97, s	17.3, CH_3_	0.94, s	17.3, CH_3_	1.18, s
20	212.4, C		212.5, C		212.3, C	
21	31.7, CH_3_	2.13, s	31.7, CH_3_	2.13, s	31.7, CH_3_	2.14, s

**^1^** Signals were overlapped with other signals.

**Table 3:** *α*-Glucosidase inhibitory activities of **1**–**4**.

Compounds	IC_50_ (μM) ^1^
**1**	>400
**2**	148.4
**3**	279.7
**4**	86.0
Acarbose ^2^	418.9

^1^ The 50% inhibitory concentration (μM). ^2^ Acarbose is used as a positive control.

## Data Availability

The data presented in the article are available in the Supplementary Materials.

## Supplementary Materials

The following are available online at: https://www.mdpi.com/article/10.3390/md21040246/s1, Figures S1–S14: ^1^H, ^13^C NMR, HSQC, COSY, HMBC, selective 1D NOESY, and HR-ESIMS data of **1** and **2**, Table S1 and Figures S15–S16: ^1^H, ^13^C NMR data, and 1D NOESY data of the reported compound, Figures S17–S20: ^1^H, ^13^C NMR, HSQC, and HR-ESIMS data of **3**, Figures S21–S28: ^1^H, ^13^C NMR, HSQC, COSY, HMBC, ROESY, 1D NOESY, and HR-ESIMS data of **4**, Figure S29: Comparison of ^1^H data of **4** in pyridine-*d*_5_ and in CD_3_OD, Figures S30–S34: ^1^H, ^13^C NMR, HSQC, COSY, and HMBC data of **5**, Figures S35–S38: ^1^H MMR, HSQC, HMBC, and HR-ESIMS data of semisynthetic **5.**
