# Supplementary material for: Thiolactones and Δ8,9-Pregnene Steroids from the Marine-Derived Fungus Meira sp. 1210CH-42 and Their α-Glucosidase Inhibitory Activity
Source: Mar Drugs. 2023 Apr 16;21(4):246. doi: 10.3390/md21040246 (PMC10140954; doi:10.3390/md21040246)
Supplement: Supplementary file 1 [file marinedrugs-21-00246-s001.zip › marinedrugs-2357466-supplementary.pdf]

*Supporting Information*

# Thiolactones and $\Delta^{8,9}$-Pregnene Steroids from the Marine-Derived Fungus *Meira* sp. 1210CH-42 and Their $\alpha$-Glucosidase Inhibitory Activity

***Corresponding Author**

Hee Jae Shin

E-mail: shinhj@kiost.ac.kr; Tel.: +82-51-664-3341; Fax: +82-51-664-3340

## Contents

Figure S1. $^{1}H$ NMR spectrum of **1** in $CD_3OD$ (600 MHz).......... 4

Figure S2. $^{13}C$ NMR spectrum of **1** in $CD_3OD$ (150 MHz). .......... 4

Figure S3. HSQC spectrum of **1** in $CD_3OD$.......... 5

Figure S4. COSY spectrum of **1** in $CD_3OD$. .......... 5

Figure S5. HMBC spectrum of **1** in $CD_3OD$.......... 6

Figure S6. Selective 1D NOESY spectrum of **1** in $CD_3OD$. .......... 7

Figure S7. HR-ESIMS spectrum of **1.**.......... 8

Figure S8. $^{1}H$ NMR spectrum of **2** in $CD_3OD$ (600 MHz). .......... 9

Figure S9. $^{13}C$ NMR spectrum of **2** in $CD_3OD$ (150 MHz). .......... 9

Figure S10. HSQC spectrum of **2** in $CD_3OD$.......... 10

Figure S11. COSY spectrum of **2** in $CD_3OD$. .......... 10

Figure S12. HMBC spectrum of **2** in $CD_3OD$.......... 11

Figure S13. Selective 1D NOESY spectrum of **2** in $CD_3OD$. .......... 12

Figure S14. HR-ESIMS spectrum of **2.**.......... 13

Figure S15. 1D NOESY spectrum of the reported compound. .......... 14

Figure S16. 1D NOESY correlations of the reported compound. .......... 14

Figure S17. $^{1}H$ NMR spectrum of **3** in $CD_3OD$ (600 MHz). .......... 15

Figure S18. $^{13}C$ NMR spectrum of **3** in $CD_3OD$ (150 MHz). .......... 15

Figure S19. HSQC spectrum of **3** in $CD_3OD$.......... 16

Figure S20. HR-ESIMS spectrum of **3**.......... 17

Figure S21. $^{1}H$ NMR spectrum of **4** in $CD_3OD$ (600 MHz). .......... 18

Figure S22. $^{13}C$ NMR spectrum of **4** in $CD_3OD$ (150 MHz). .......... 18

Figure S23. HSQC spectrum of **4** in $CD_3OD$. 19

Figure S24. COSY spectrum of **4** in $CD_3OD$. 19

Figure S25. HMBC spectrum of **4** in $CD_3OD$. 20

Figure S26. ROESY spectrum of **4** in $CD_3OD$. 20

Figure S27. Selective 1D NOESY spectrum of **4** in $CD_3OD$. 21

Figure S28. HR-ESIMS spectrum of **4**. 22

Figure S29. Comparison of $^1H$ NMR spectra of **4** in pyridine-$d_5$ and in $CD_3OD$. 23

Figure S30. $^1H$ NMR spectrum of **5** in $CD_3OD$ (600 MHz) 24

Figure S31. $^{13}C$ NMR spectrum of **5** in $CD_3OD$ (150 MHz). 24

Figure S32. HSQC spectrum of **5** in $CD_3OD$. 25

Figure S33. COSY spectrum of **5** in $CD_3OD$. 25

Figure S34. HMBC spectrum of **5** in $CD_3OD$. 26

Figure S35. Comparison of $^1H$ NMR spectra between natural **5** and semisynthetic **5** in $CD_3OD$ (600 MHz). 27

Figure S36. HSQC spectrum of semisynthetic **5** in $CD_3OD$. 28

Figure S37. HMBC spectrum of semisynthetic **5** in $CD_3OD$. 28

Figure S38. HR-ESIMS spectrum of semisynthetic **5**. 29

Table S1. $^1H$ and $^{13}C$ NMR data of **1**, **2**, and the reported compound in $CDCl_3$ 14

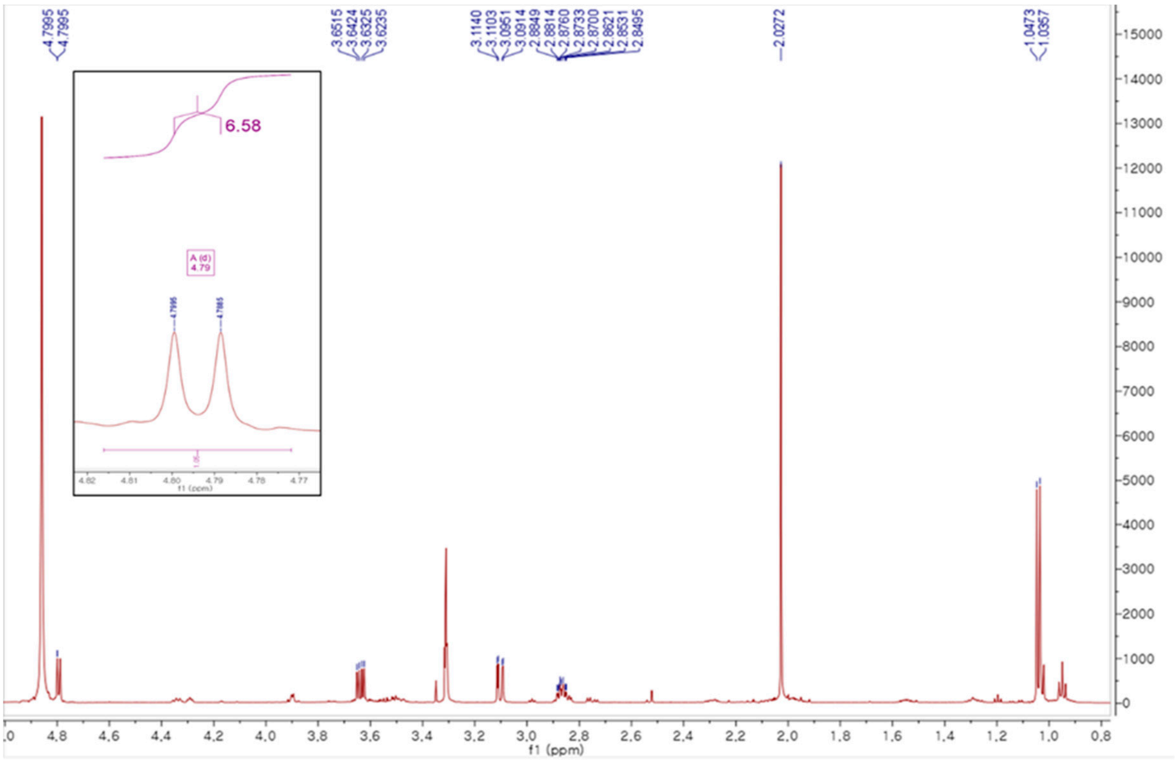


Figure S1. $^{1}$H NMR spectrum of **1** in $CD_3OD$ (600 MHz).

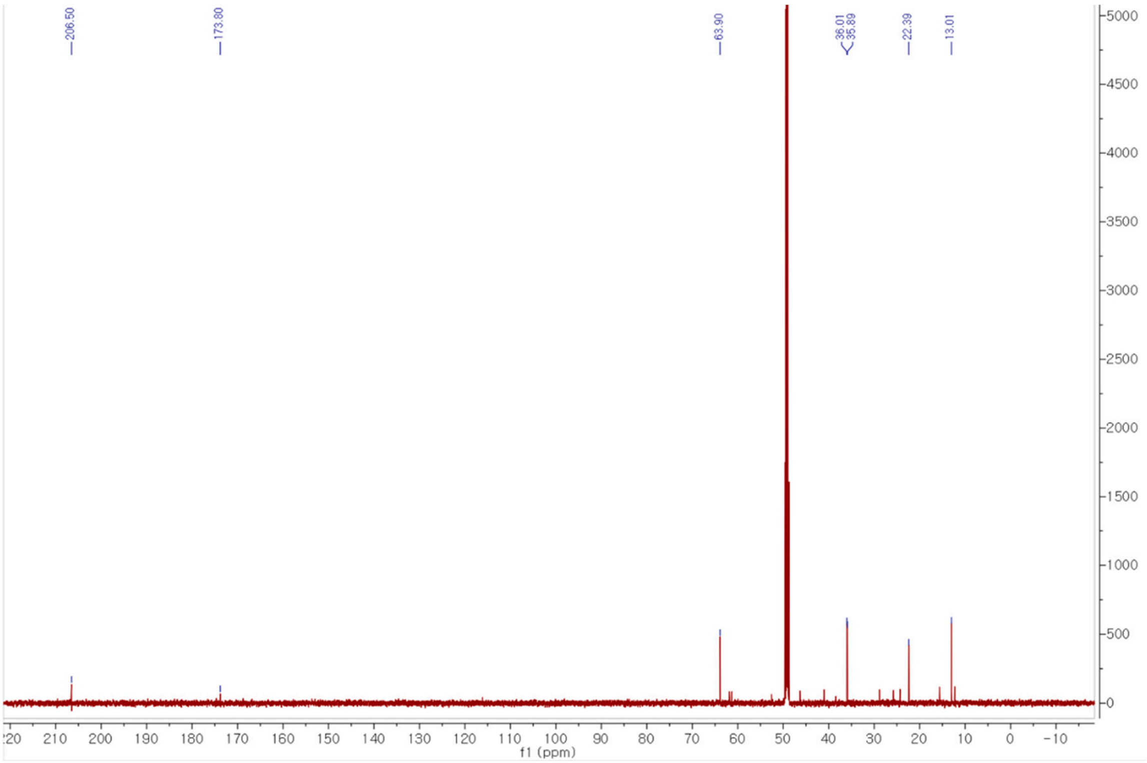


Figure S2. $^{13}$C NMR spectrum of **1** in $CD_3OD$ (150 MHz).

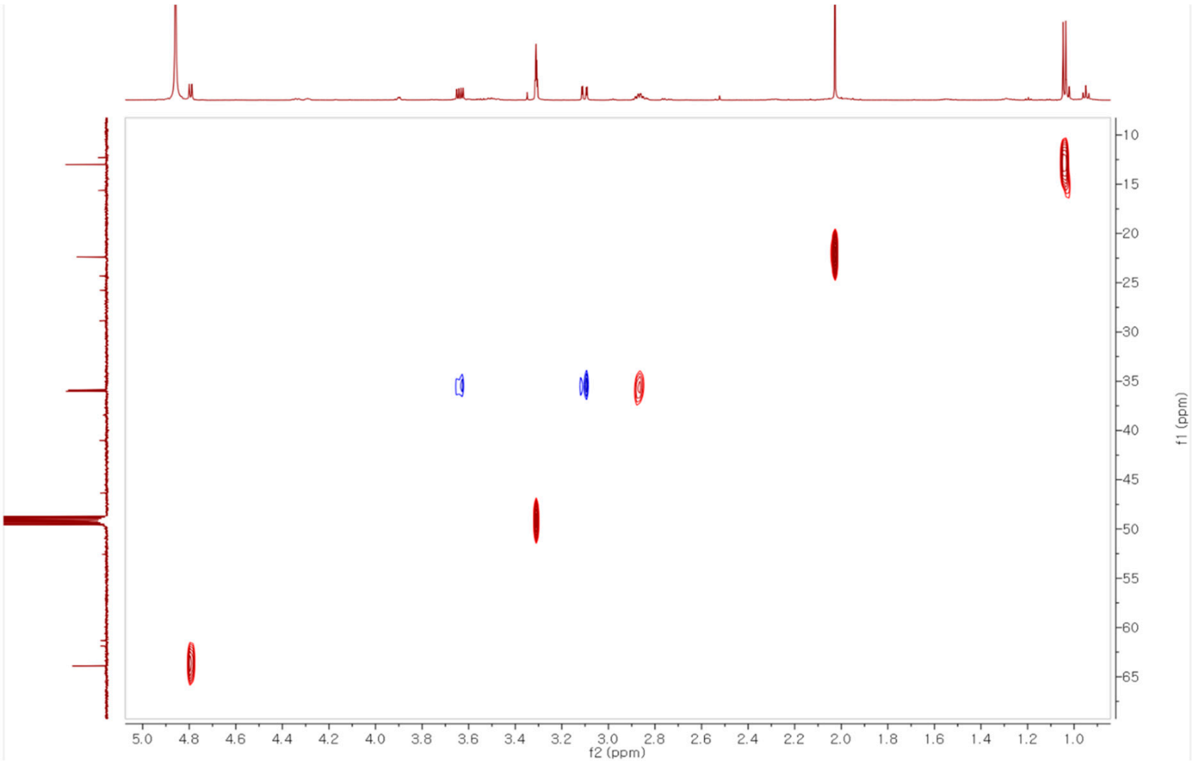


Figure S3. HSQC spectrum of **1** in $CD_3OD$.

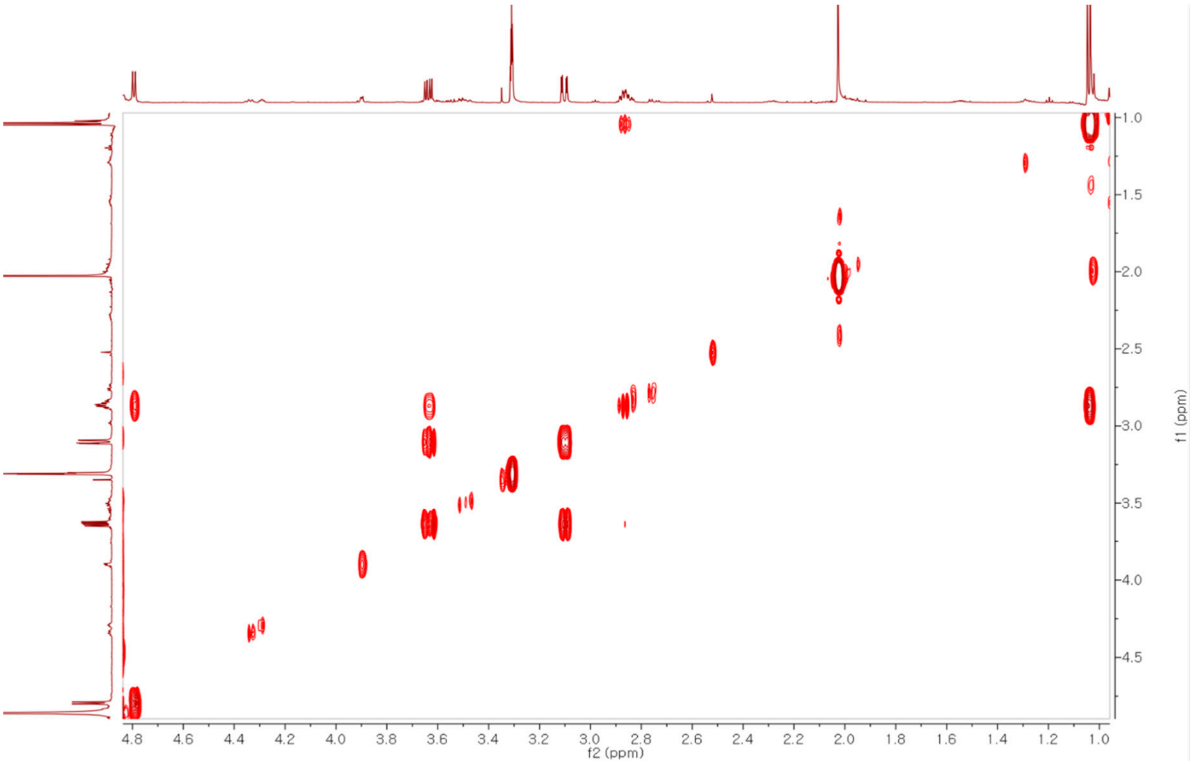


Figure S4. COSY spectrum of **1** in $CD_3OD$.

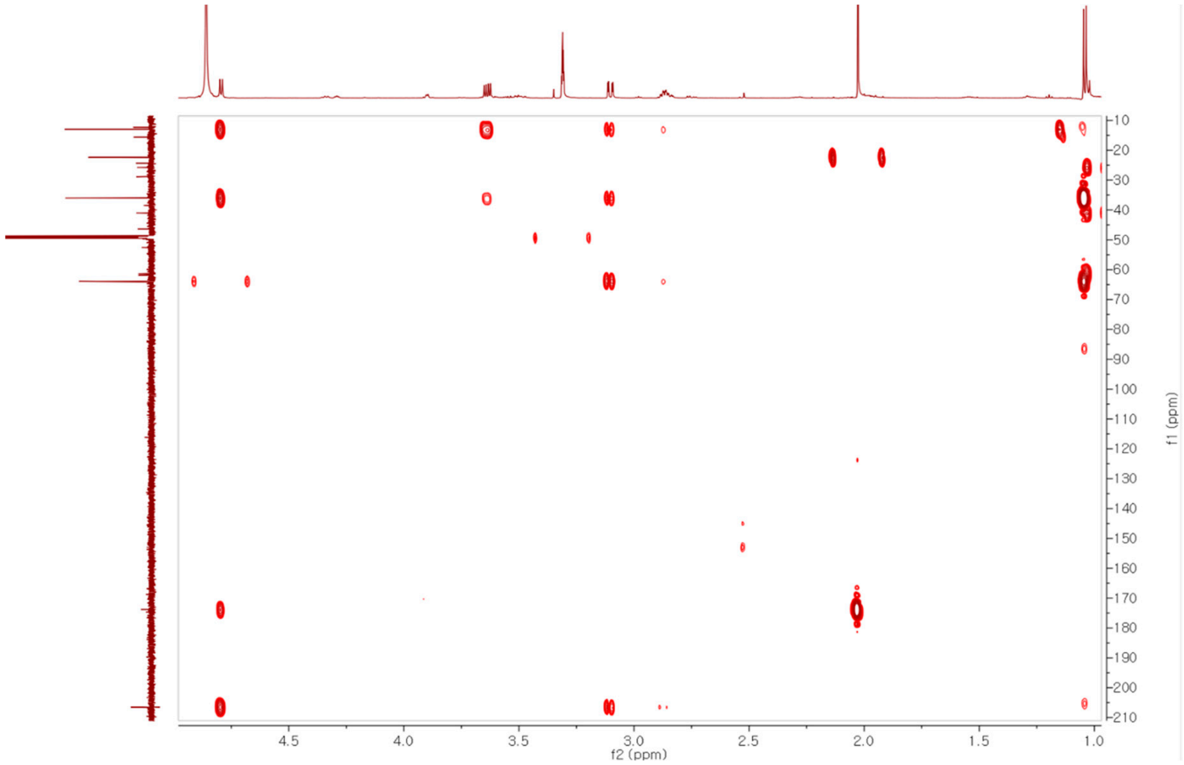


Figure S5. HMBC spectrum of **1** in $CD_3OD$.

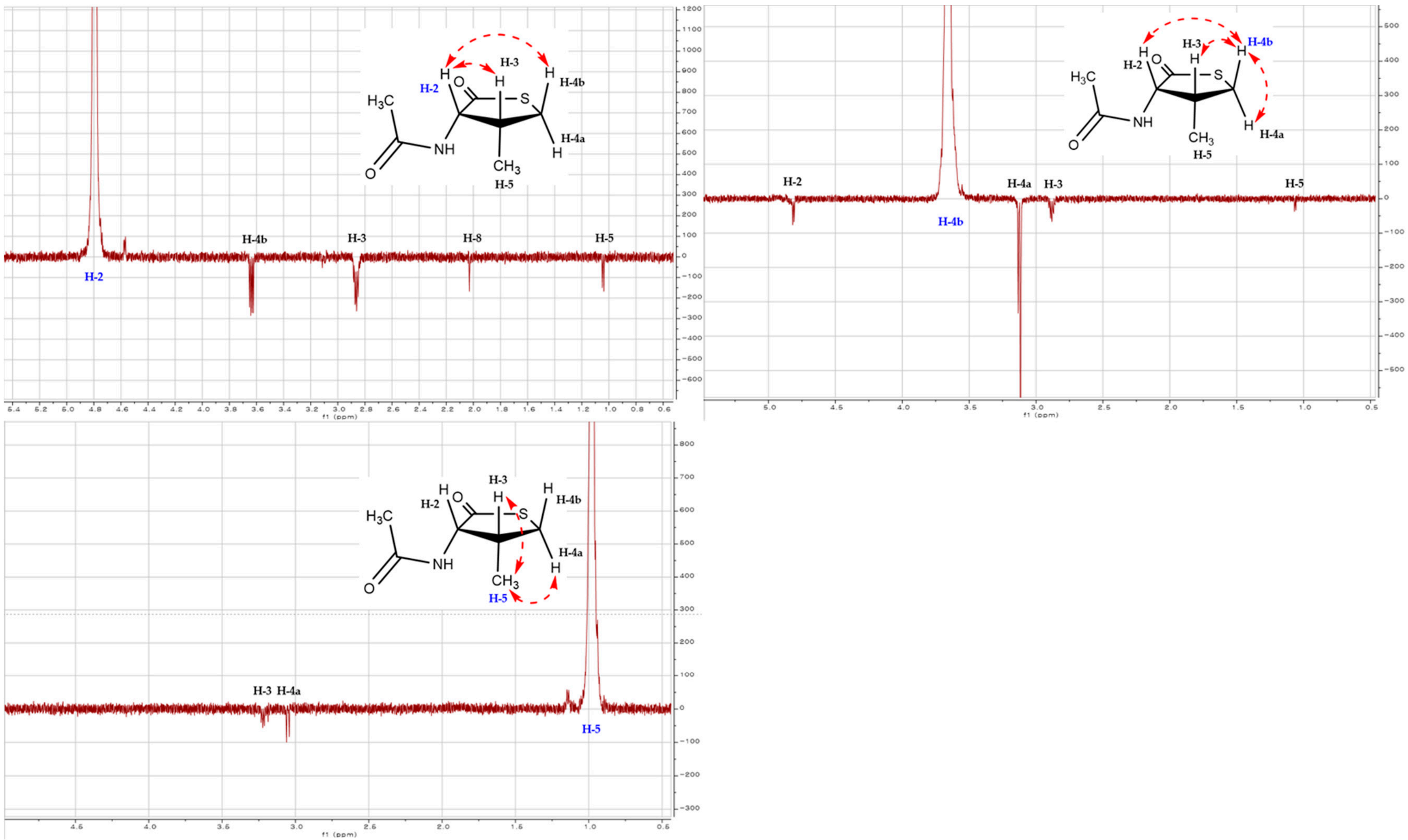


Figure S6. Selective 1D NOESY spectrum of **1** in $CD_3OD$.

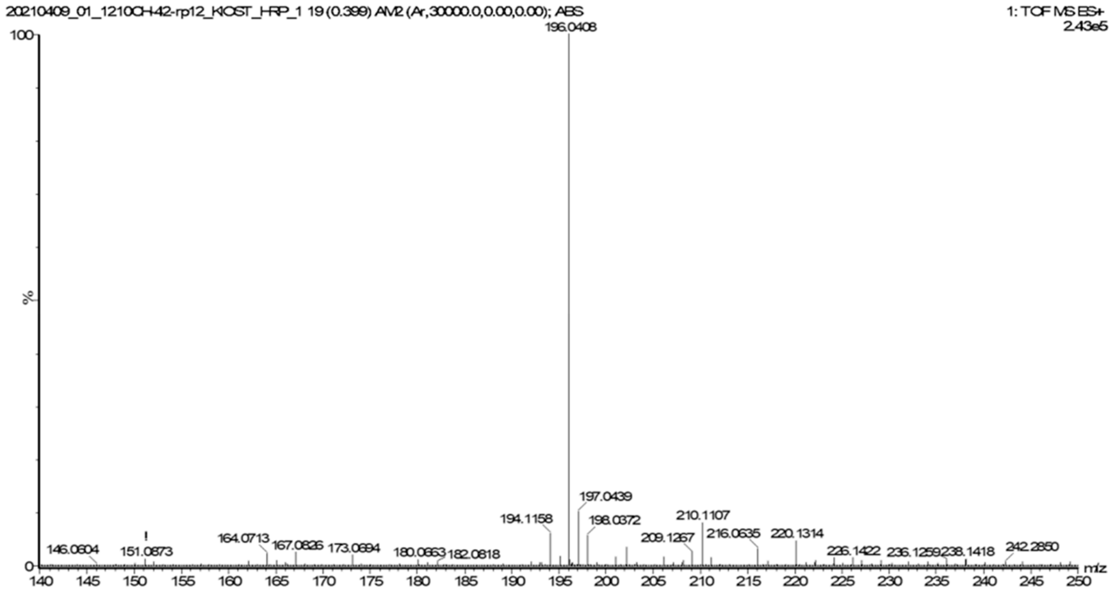


**Elemental Composition Report**

Single Mass Analysis
Tolerance = 5.0 PPM / DBE: min = -1.5, max = 100.0
Element prediction: Off
Number of isotope peaks used for i-FIT = 3

Monoisotopic Mass, Even Electron Ions
200 formula(e) evaluated with 2 results within limits (all results (up to 1000) for each mass)
Elements Used:
C: 0-25 H: 0-30 N: 0-1 O: 0-2 Na: 0-1 S: 0-2

| | | | | | | | | |
|---|---|---|---|---|---|---|---|---|
| Minimum: | | | | -1.5 | | | | |
| Maximum: | | 100.0 | 5.0 | 100.0 | | | | |
| Mass | Calc. Mass | mDa | PPM | DBE | i-FIT | Norm | Conf(%) | Formula |
| 196.0408 | 196.0408 | 0.0 | 0.0 | 2.5 | 1312.0 | 0.000 | 100.00 | C7 H11 N O2 Na S |
| | 196.0399 | 0.9 | 4.6 | 10.5 | 1327.8 | 15.758 | 0.00 | C12 H6 N O2 |

Figure S7. HR-ESIMS spectrum of **1.**

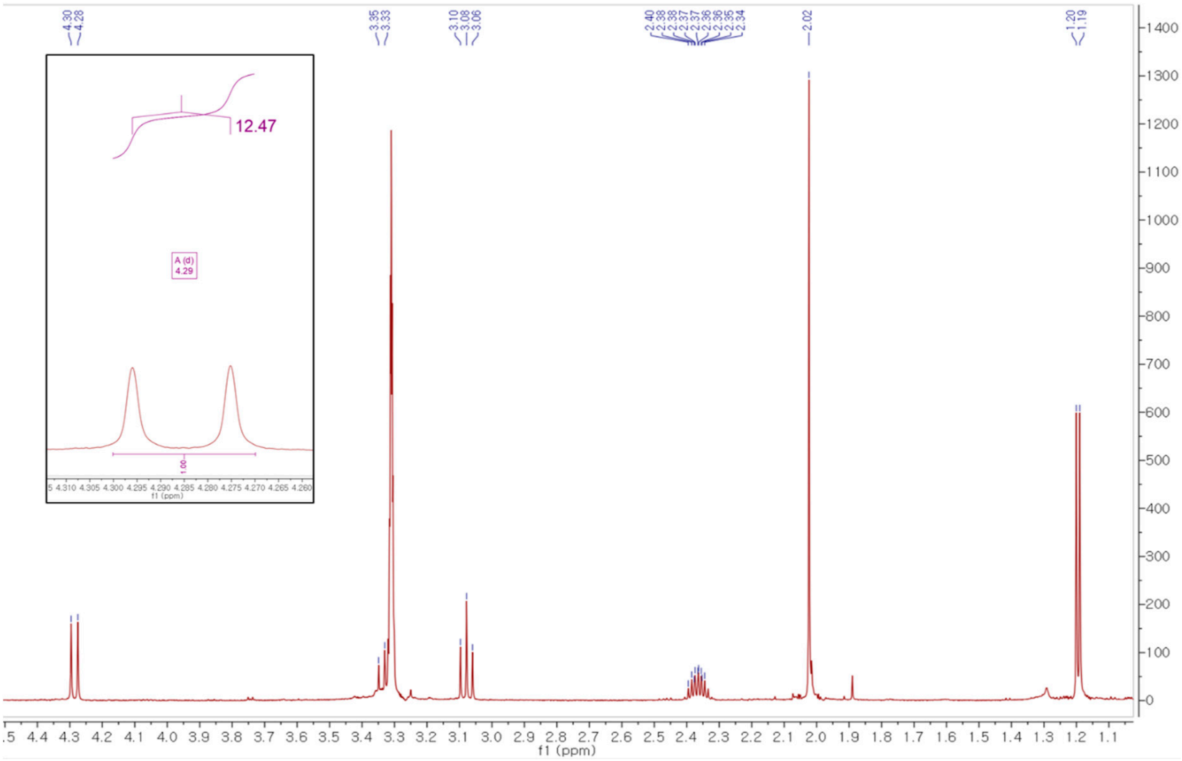

Figure S8. $^{1}$H NMR spectrum of **2** in $CD_3OD$ (600 MHz).

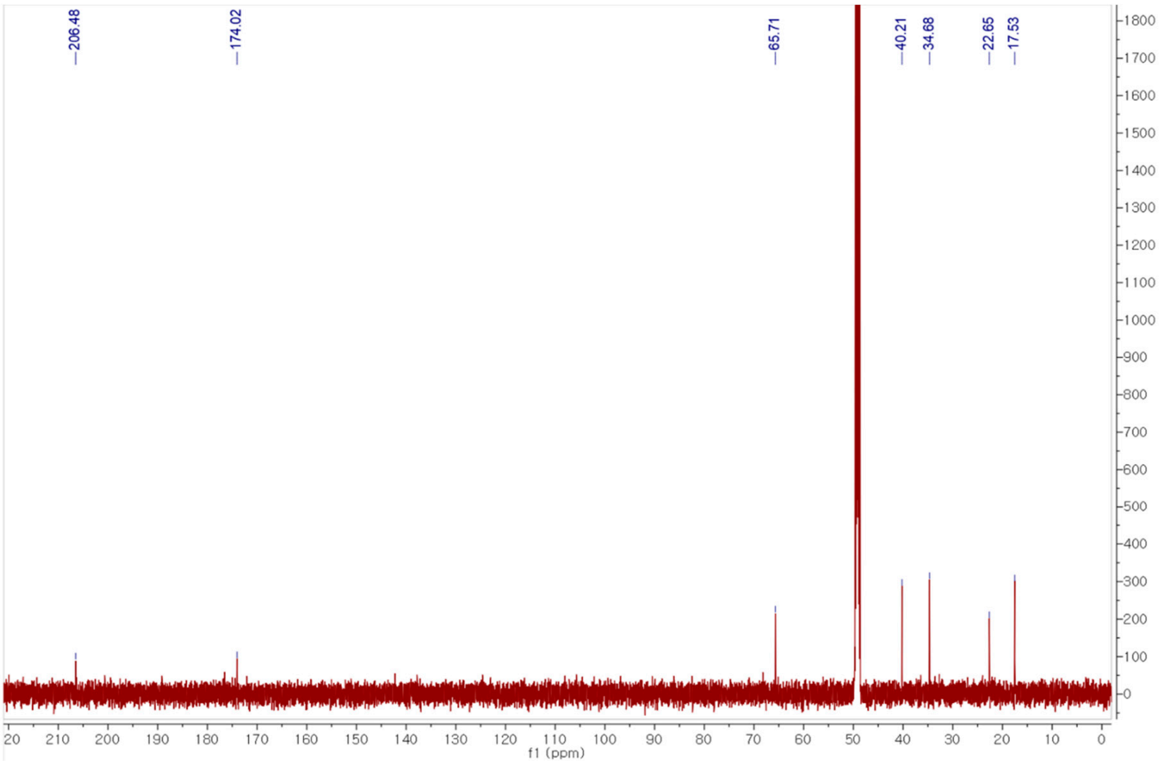


Figure S9. $^{13}$C NMR spectrum of **2** in $CD_3OD$ (150 MHz).

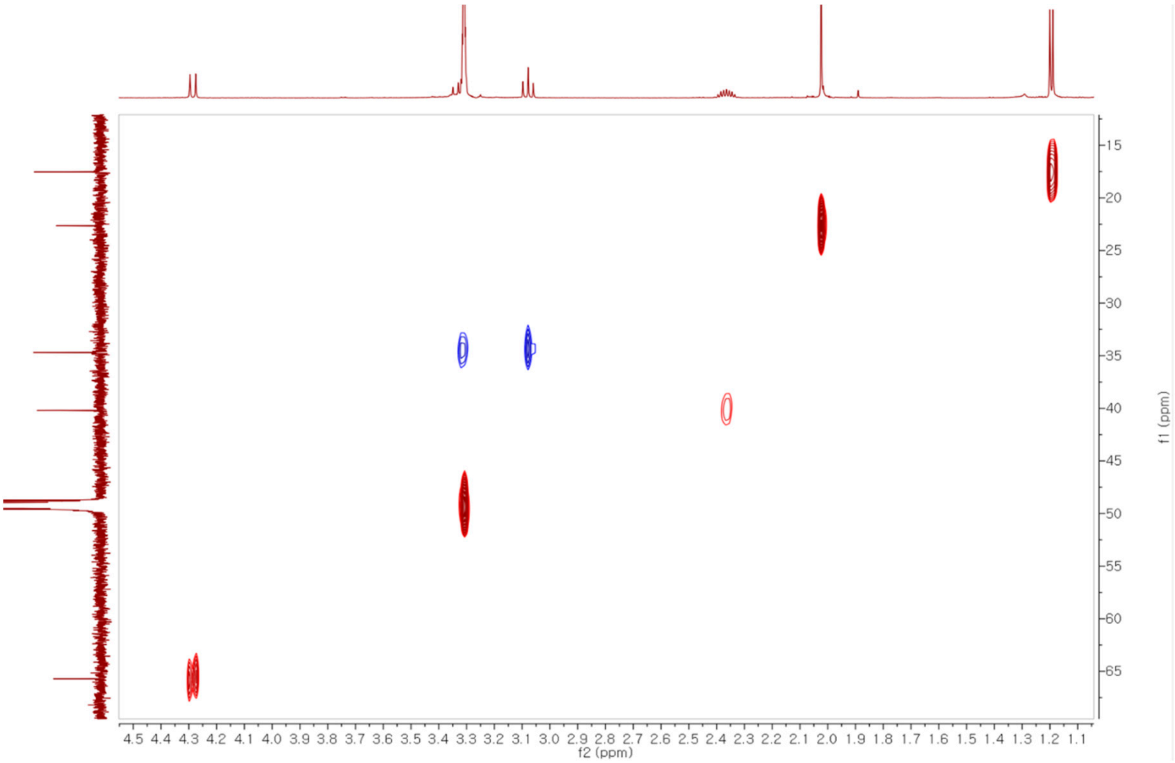


Figure S10. HSQC spectrum of **2** in $CD_3OD$.

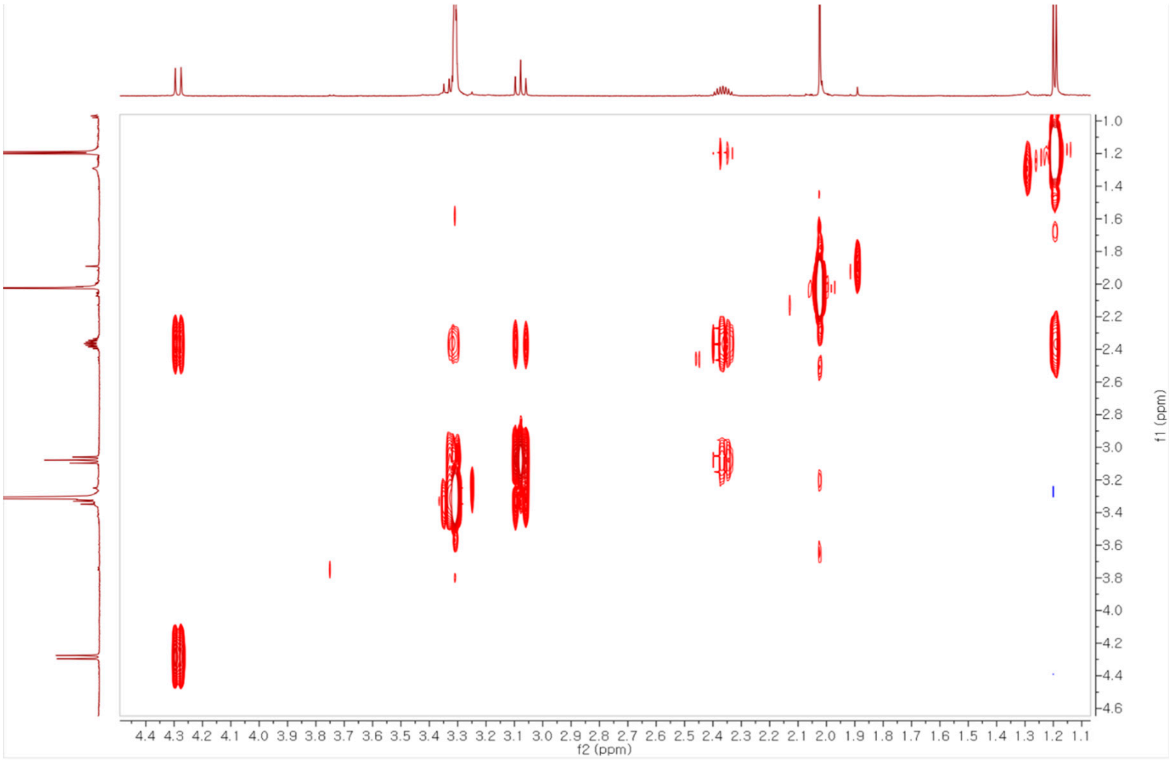


Figure S11. COSY spectrum of **2** in $CD_3OD$.

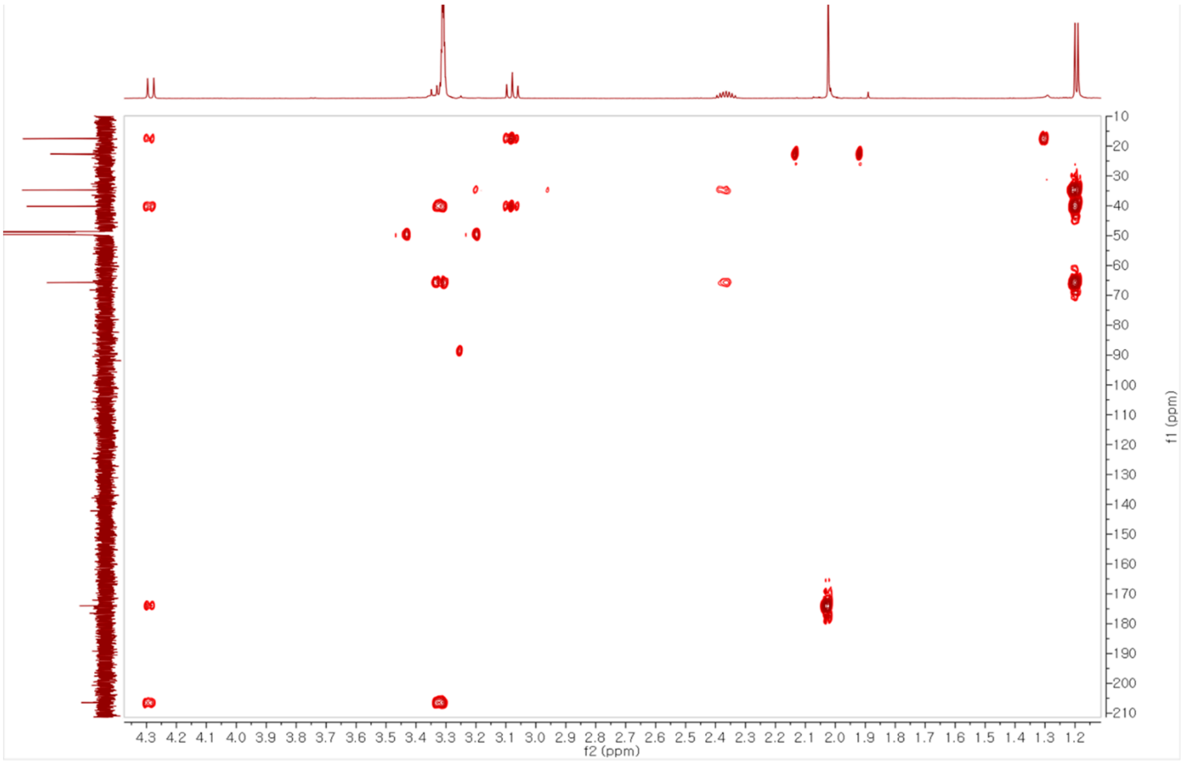


Figure S12. HMBC spectrum of **2** in $CD_3OD$.

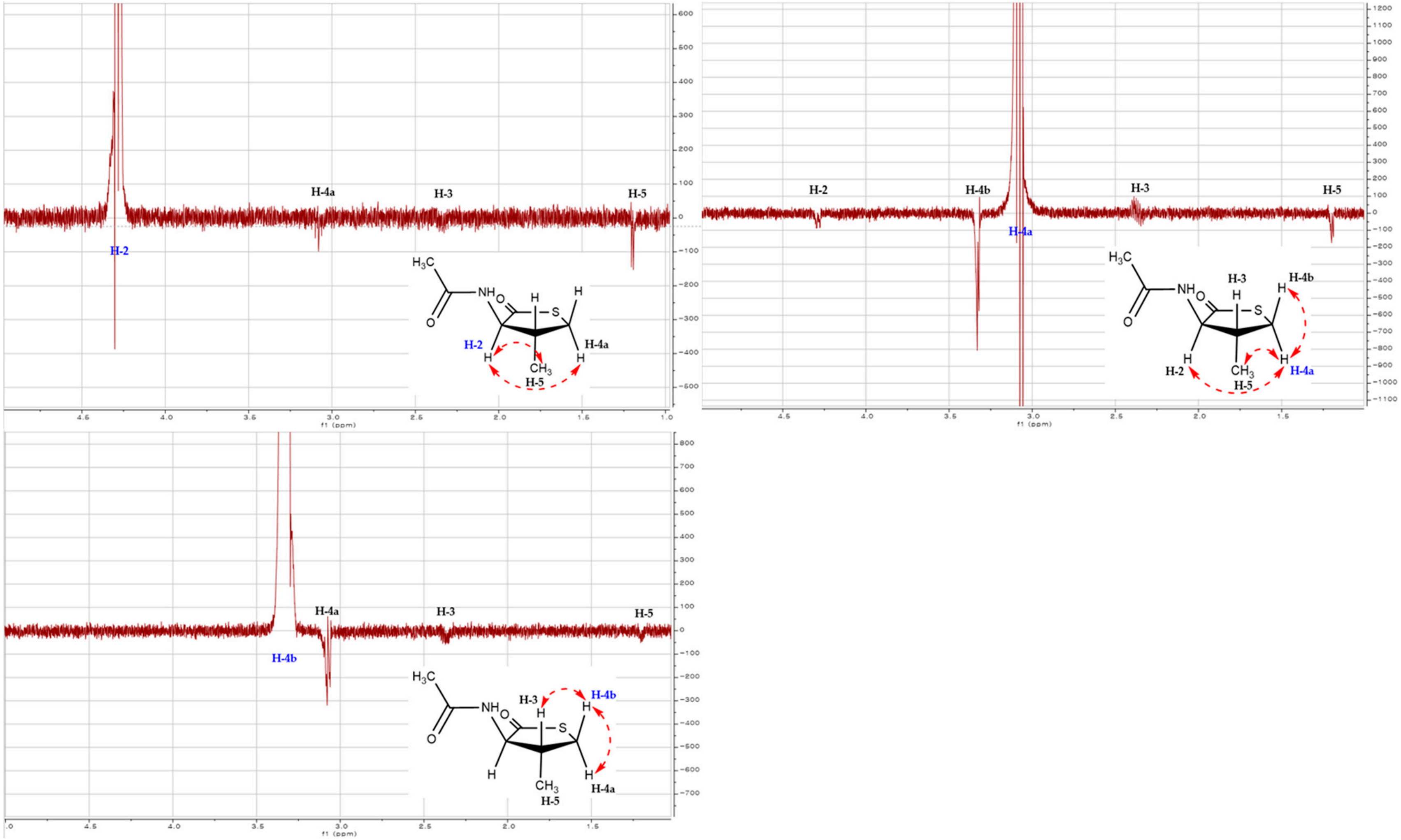


Figure S13. Selective 1D NOESY spectrum of **2** in CD3OD.

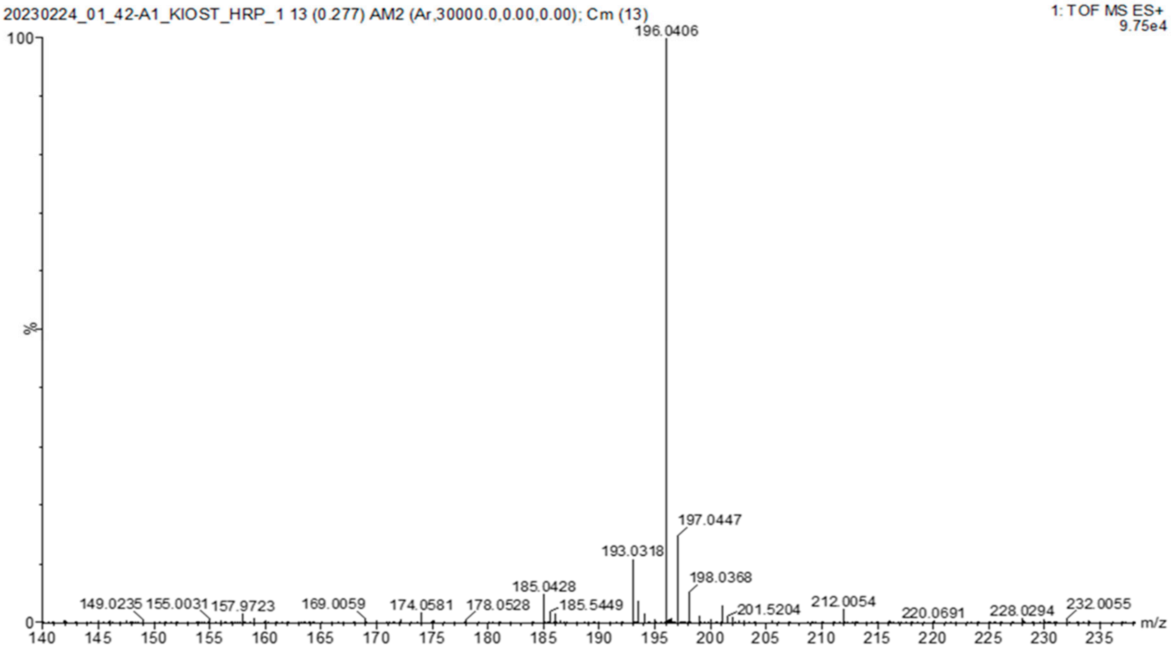


**Elemental Composition Report**

Single Mass Analysis
Tolerance = 5.0 PPM / DBE: min = -9.0, max = 100.0
Element prediction: Off
Number of isotope peaks used for i-FIT = 3

Monoisotopic Mass, Even Electron Ions
285 formula(e) evaluated with 2 results within limits (up to 50 closest results for each mass)
Elements Used:
C: 0-25 H: 0-100 N: 0-3 O: 0-4 Na: 0-1 S: 0-1

| | | | | | | | | |
|---|---|---|---|---|---|---|---|---|
| Minimum: | | | | -9.0 | | | | |
| Maximum: | | 5.0 | 5.0 | 100.0 | | | | |
| Mass | Calc. Mass | mDa | PPM | DBE | i-FIT | Norm | Conf(%) | Formula |
| 196.0406 | 196.0408 | -0.2 | -1.0 | 2.5 | 974.6 | 0.122 | 88.48 | C7 H11 N O2 Na S |
| | 196.0399 | 0.7 | 3.6 | 10.5 | 976.7 | 2.161 | 11.52 | C12 H6 N O2 |

Figure S14. HR-ESIMS spectrum of **2.**

Table S1. $^{1}$H and $^{13}$C NMR data of **1**, **2**, and the reported compound in $CDCl_3$

| Position | **1** | | **2** | | Reported compound* | |
|---|---|---|---|---|---|---|
| | $\delta_C$, type | $\delta_H$ ($J$ in Hz) | $\delta_C$, type | $\delta_H$ ($J$ in Hz) | $\delta_C$, type | $\delta_H$ ($J$ in Hz) |
| **1** | 205.3, C | | 204.9, C | | 204.9, C | |
| **2** | 63.9, CH | 4.57, ddd (6.7, 5.4, 1.4) | 64.4, CH | 4.45, dd (12.5, 8.6) | 64.4, CH | 4.45, d (12.5, 8.7) |
| **3** | 34.5, CH | 3.20, m | 40.5, CH | 2.24, m | 40.5, CH | 2.37, m |
| **4a** | 35.2, $CH_2$ | 3.05, d (11.5) | 34.0, $CH_2$ | 3.02, t (11.2) | 34.0, $CH_2$ | 3.02, t |
| **4b** | | 3.61, dd (11.5, 5.2) | | 3.24, dd (11.2, 6.2) | | 3.24, dd (11.2, 6.2) |
| **5** | 12.2, $CH_3$ | 0.97, d (7.0) | 17.5, $CH_3$ | 1.25, d (6.6) | 17.5, $CH_3$ | 1.25, d (6.5) |
| **6, -NH** | | 5.94, s | | 5.66, d (8.6) | | 5.67, s |
| **7** | 170.6, C | | 170.9, C | | 170.9, C | |
| **8** | 23.1, $CH_3$ | 2.07, s | 23.4, $CH_3$ | 2.09, s | 23.4, $CH_3$ | 2.08, s |

* ***Nat. Prod. Res.*** **2020, *34*, 2926-2930.**

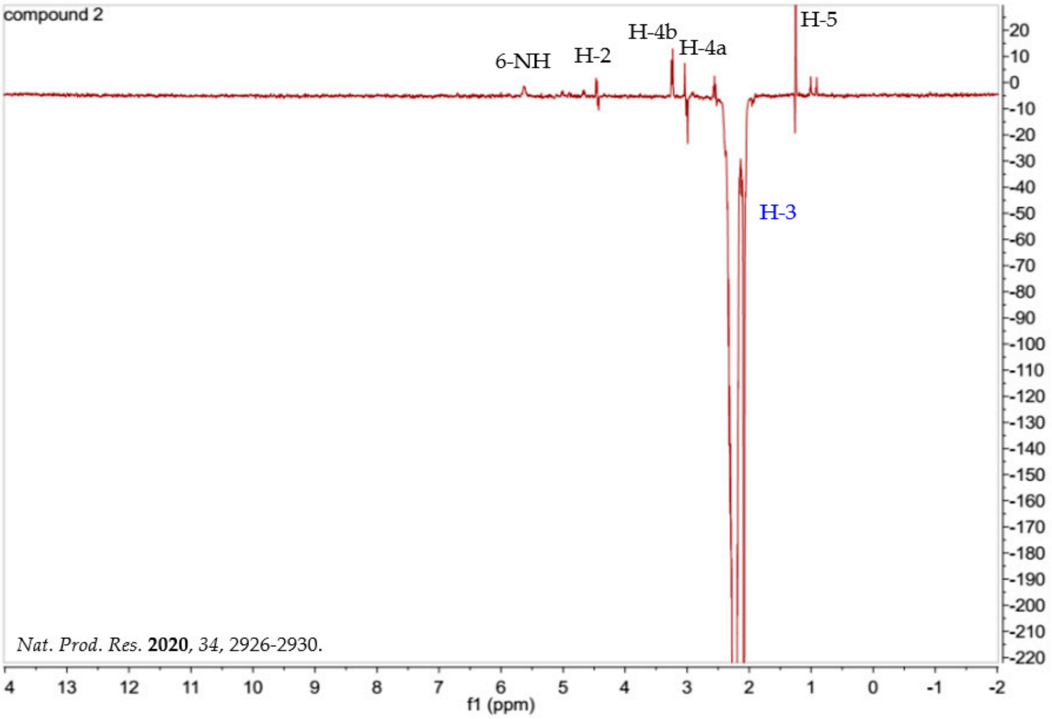


Figure S15. 1D NOESY spectrum of the reported compound.

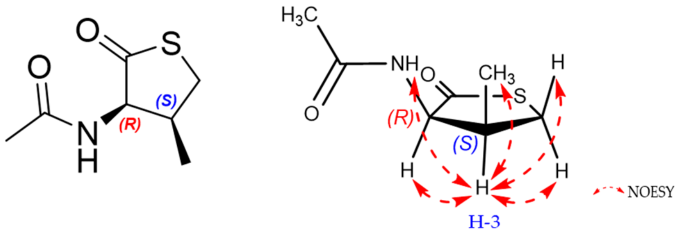


Figure S16. 1D NOESY correlations of the reported compound.

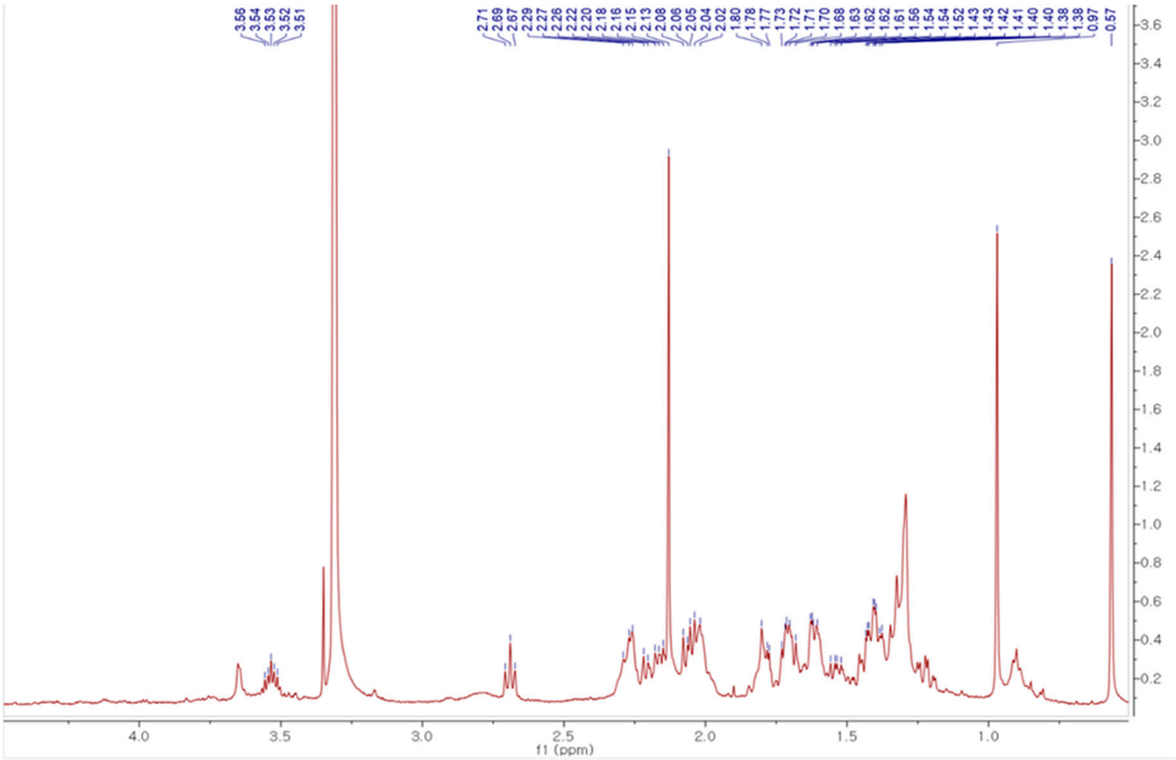

Figure S17. $^{1}H$ NMR spectrum of **3** in $CD_3OD$ (600 MHz).

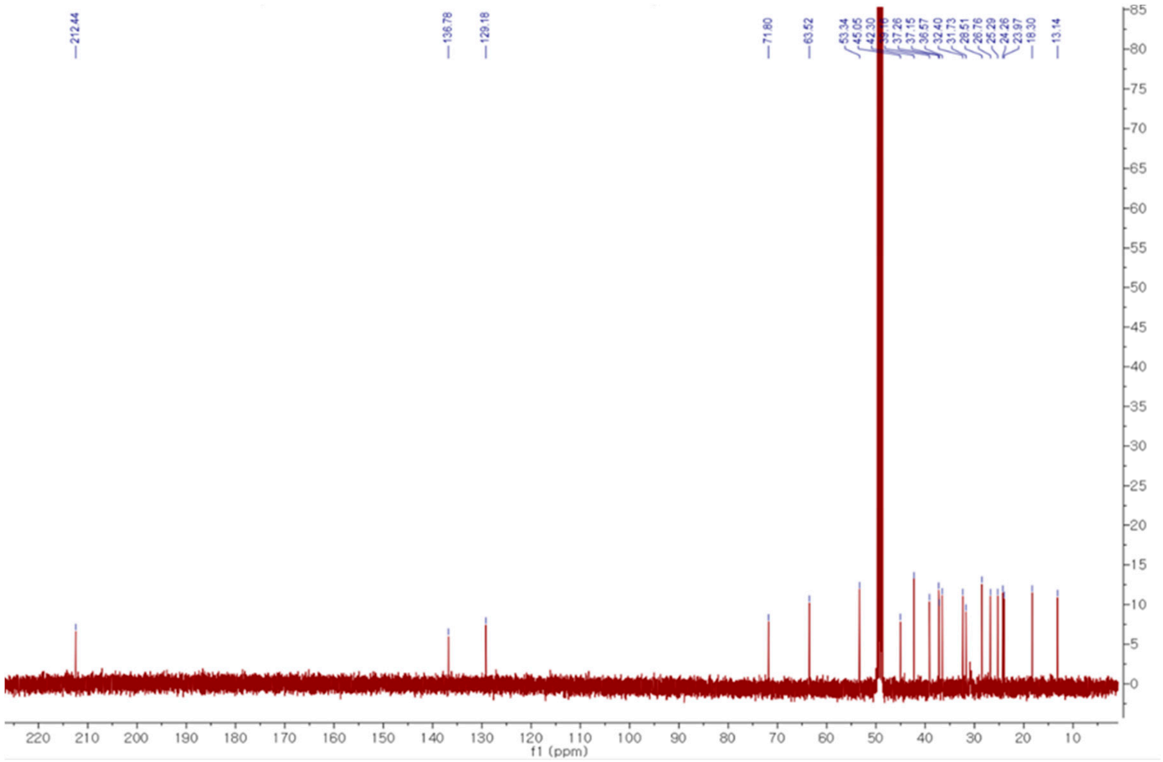

Figure S18. $^{13}C$ NMR spectrum of **3** in $CD_3OD$ (150 MHz).

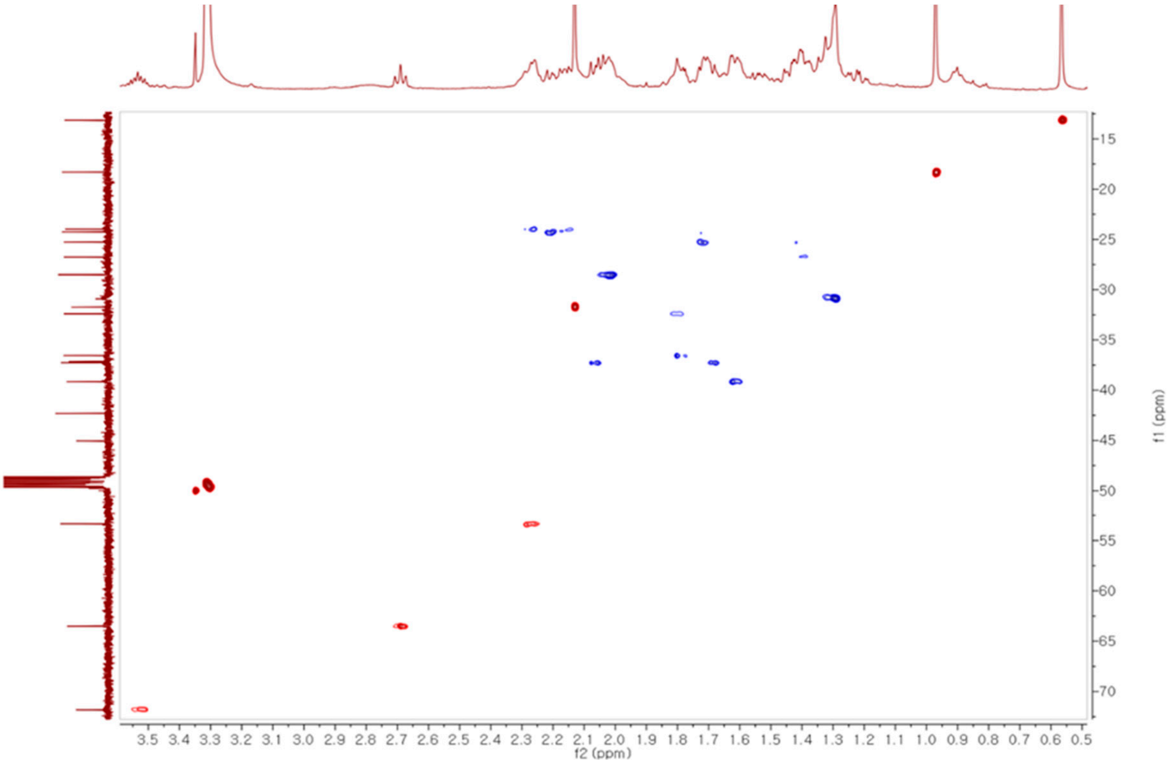


Figure S19. HSQC spectrum of **3** in $CD_3OD$.

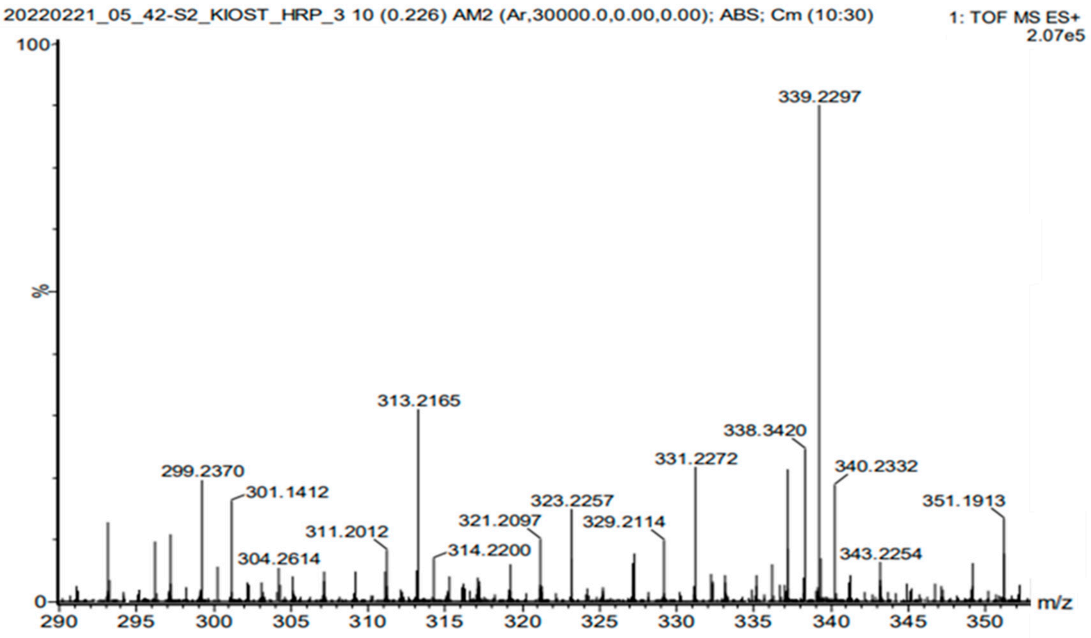


**Elemental Composition Report**

Single Mass Analysis
Tolerance = 5.0 PPM / DBE: min = -3.0, max = 200.0
Element prediction: Off
Number of isotope peaks used for i-FIT = 3

Monoisotopic Mass, Even Electron Ions
68 formula(e) evaluated with 1 results within limits (all results (up to 1000) for each mass)
Elements Used:
C: 0-31 H: 0-50 O: 0-7 Na: 0-1

| | | | | | | | | |
|---|---|---|---|---|---|---|---|---|
| Minimum: | | | | -3.0 | | | | |
| Maximum: | | 300.0 | 5.0 | 200.0 | | | | |
| Mass | Calc. Mass | mDa | PPM | DBE | i-FIT | Norm | Conf(%) | Formula |
| 339.2297 | 339.2300 | -0.3 | -0.9 | 5.5 | 905.4 | n/a | n/a | C21 H32 O2 Na |

Figure S20. HR-ESIMS spectrum of **3**.

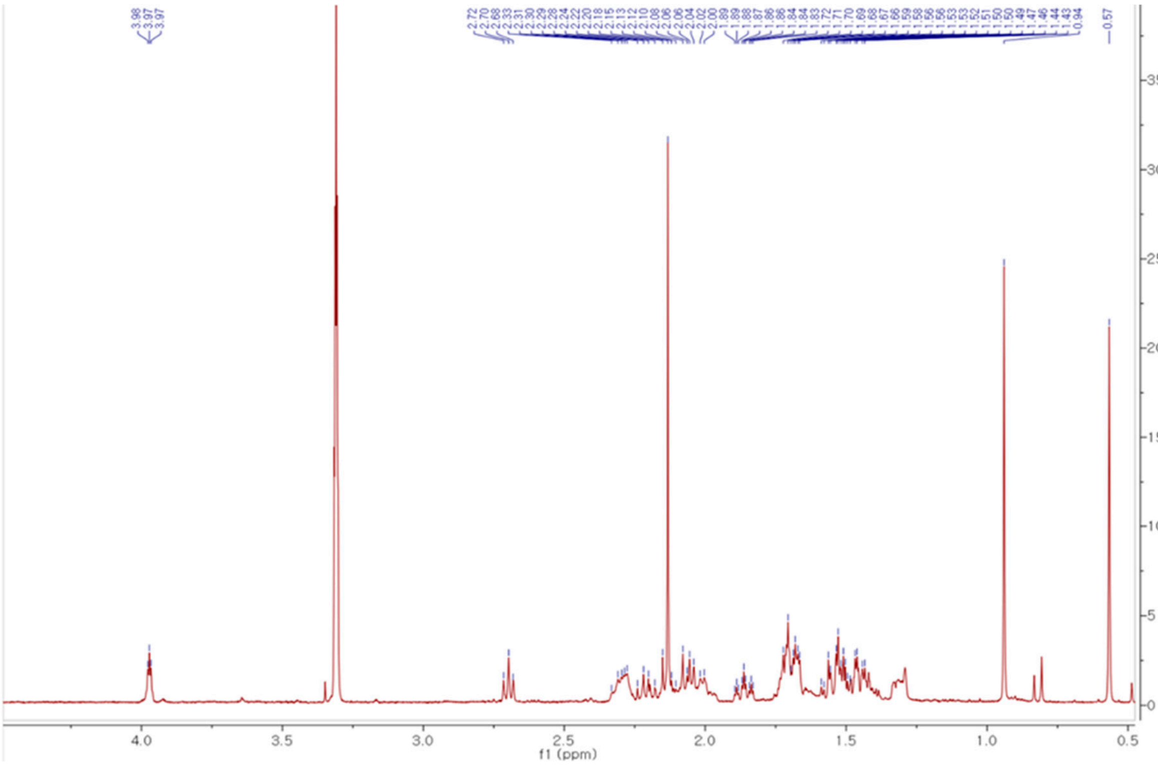


Figure S21. $^{1}H$ NMR spectrum of **4** in $CD_3OD$ (600 MHz).

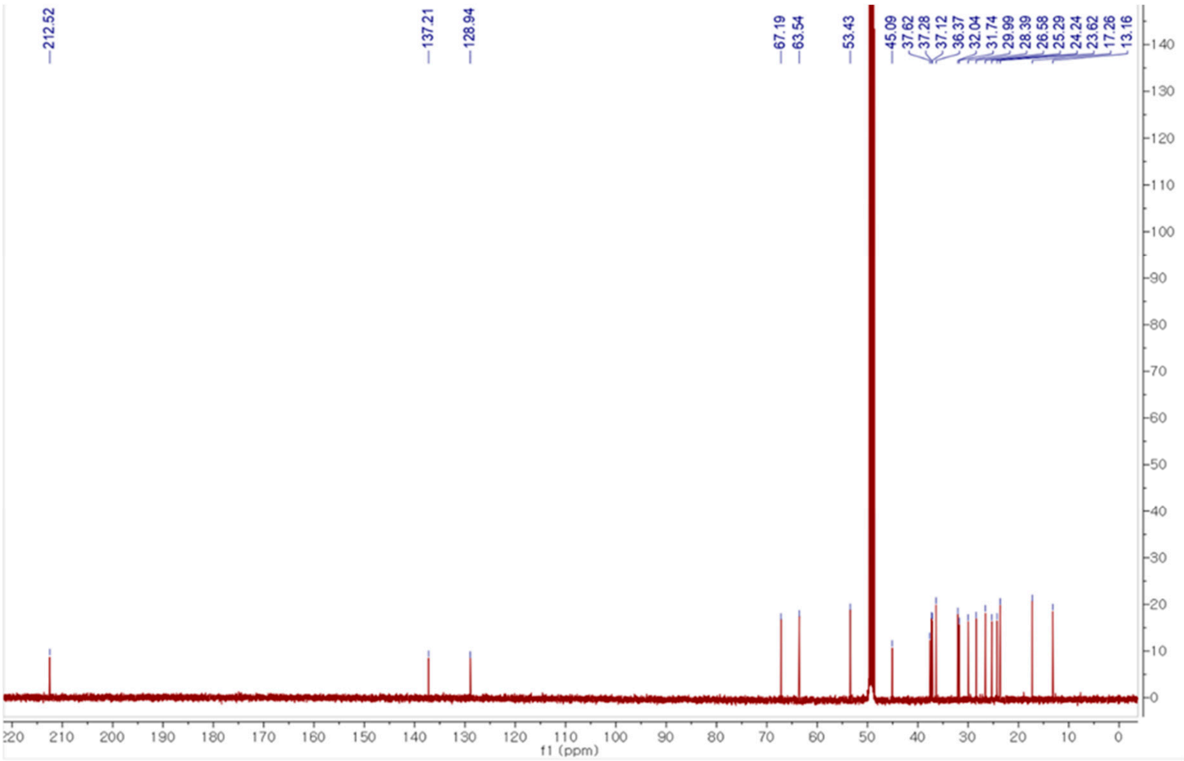


Figure S22. $^{13}C$ NMR spectrum of **4** in $CD_3OD$ (150 MHz).

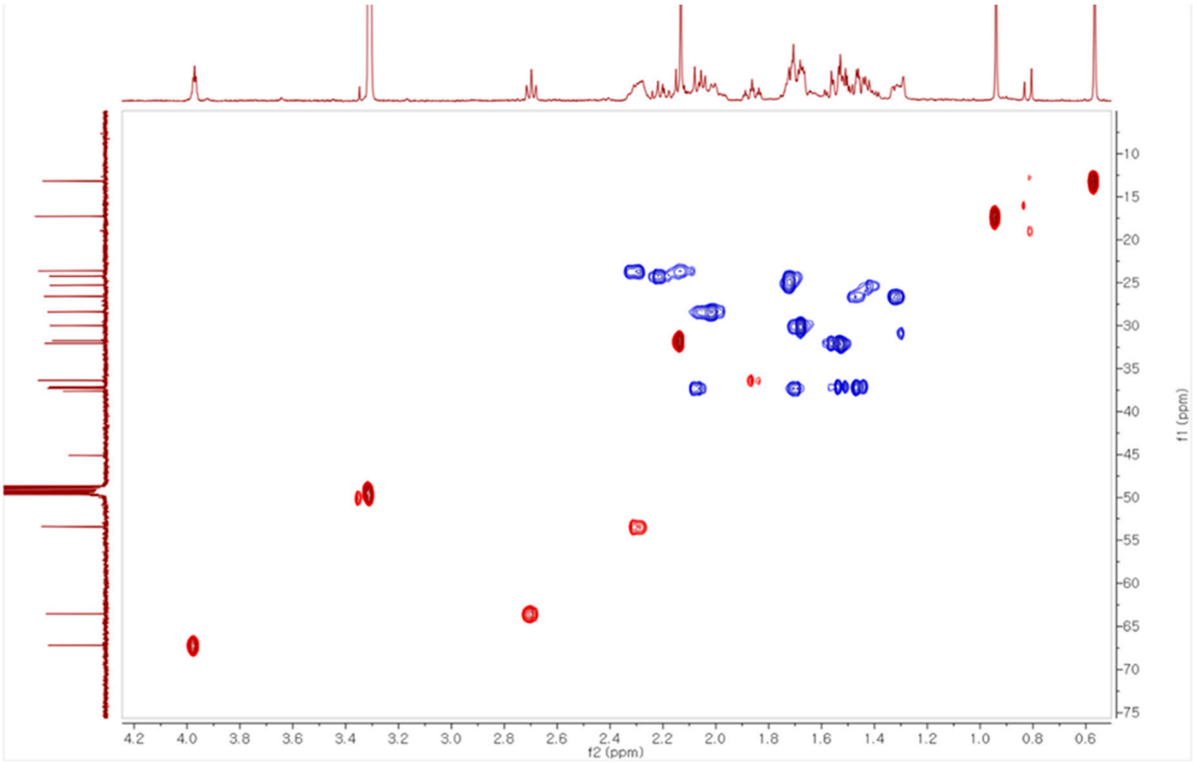


Figure S23. HSQC spectrum of **4** in $CD_3OD$.

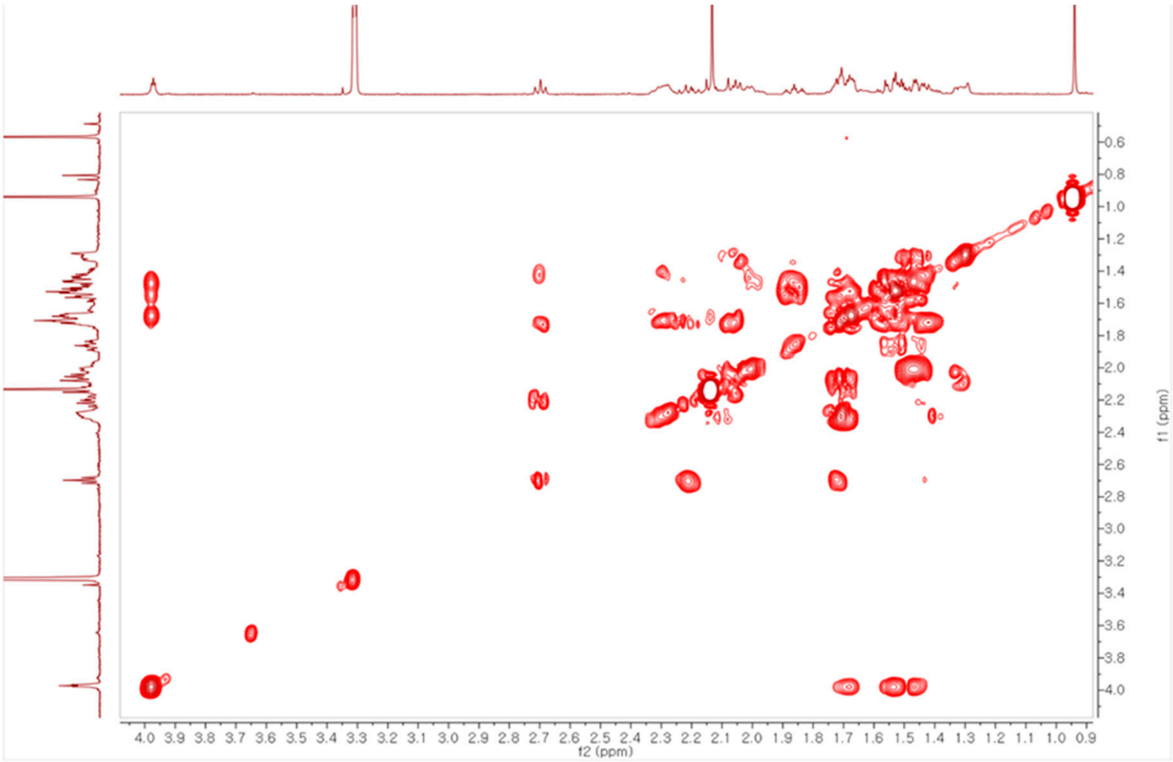


Figure S24. COSY spectrum of **4** in $CD_3OD$.

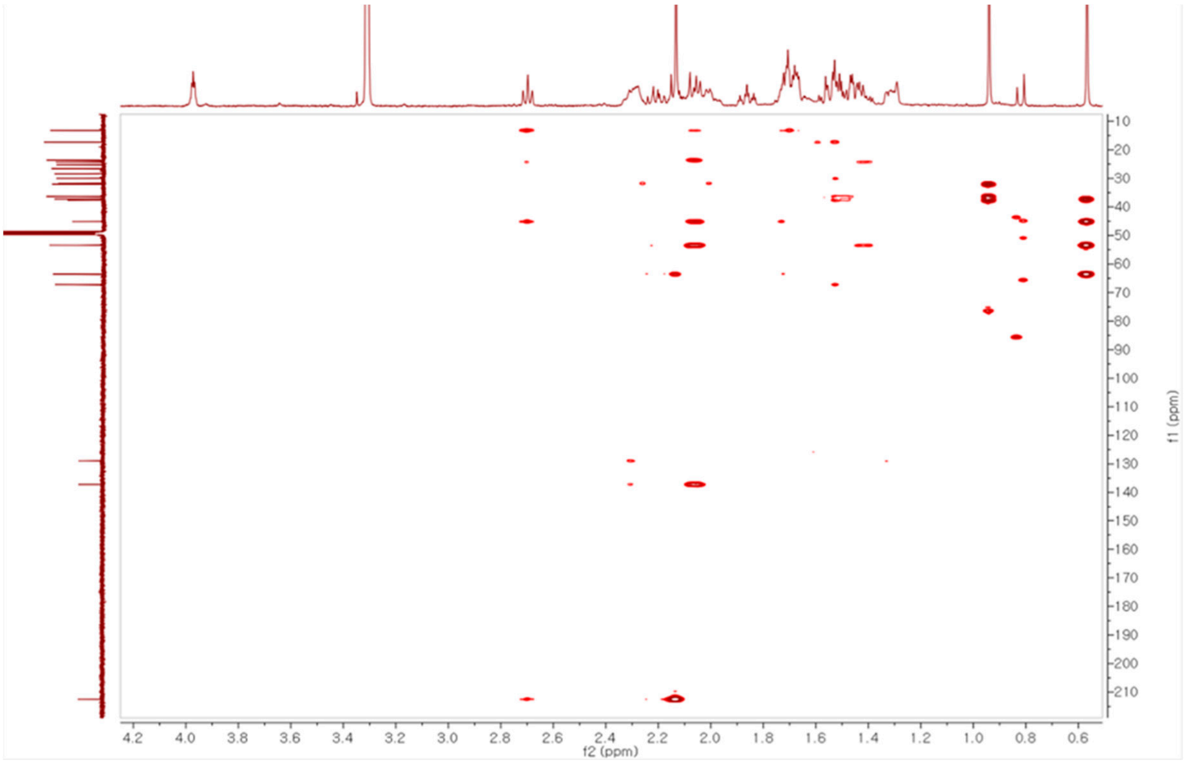


Figure S25. HMBC spectrum of **4** in $CD_3OD$.

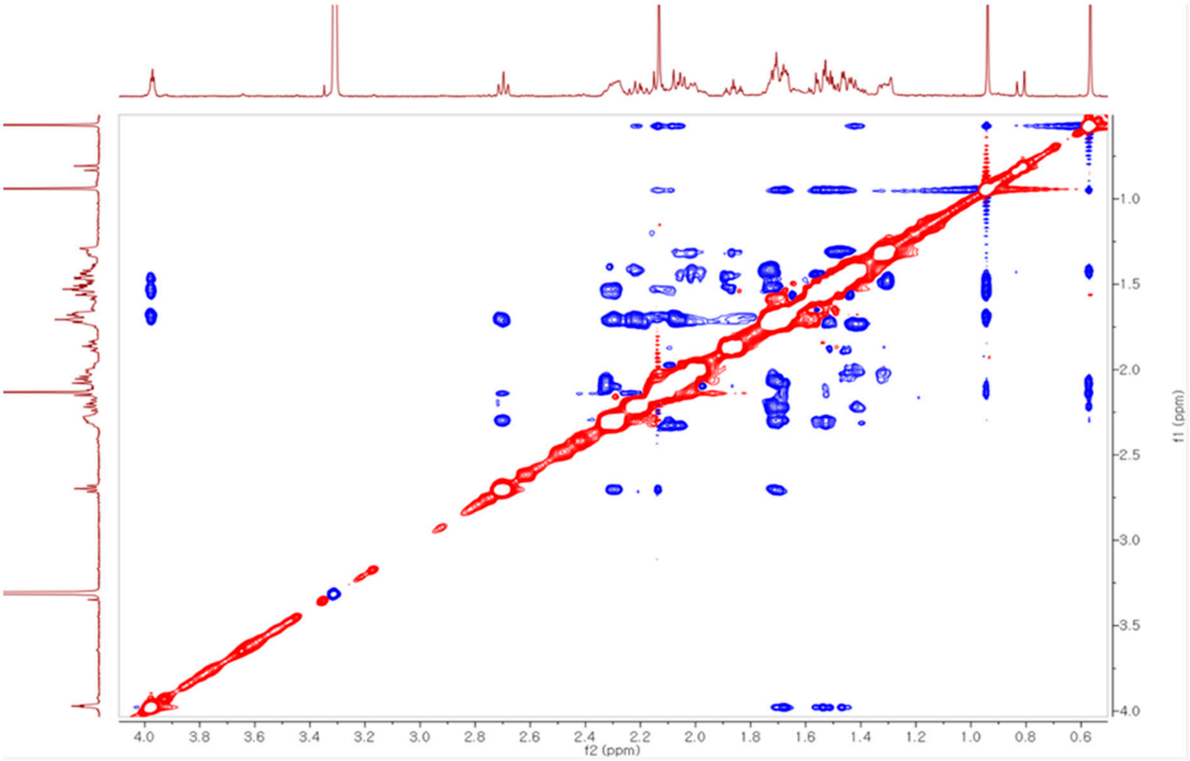


Figure S26. ROESY spectrum of **4** in $CD_3OD$.

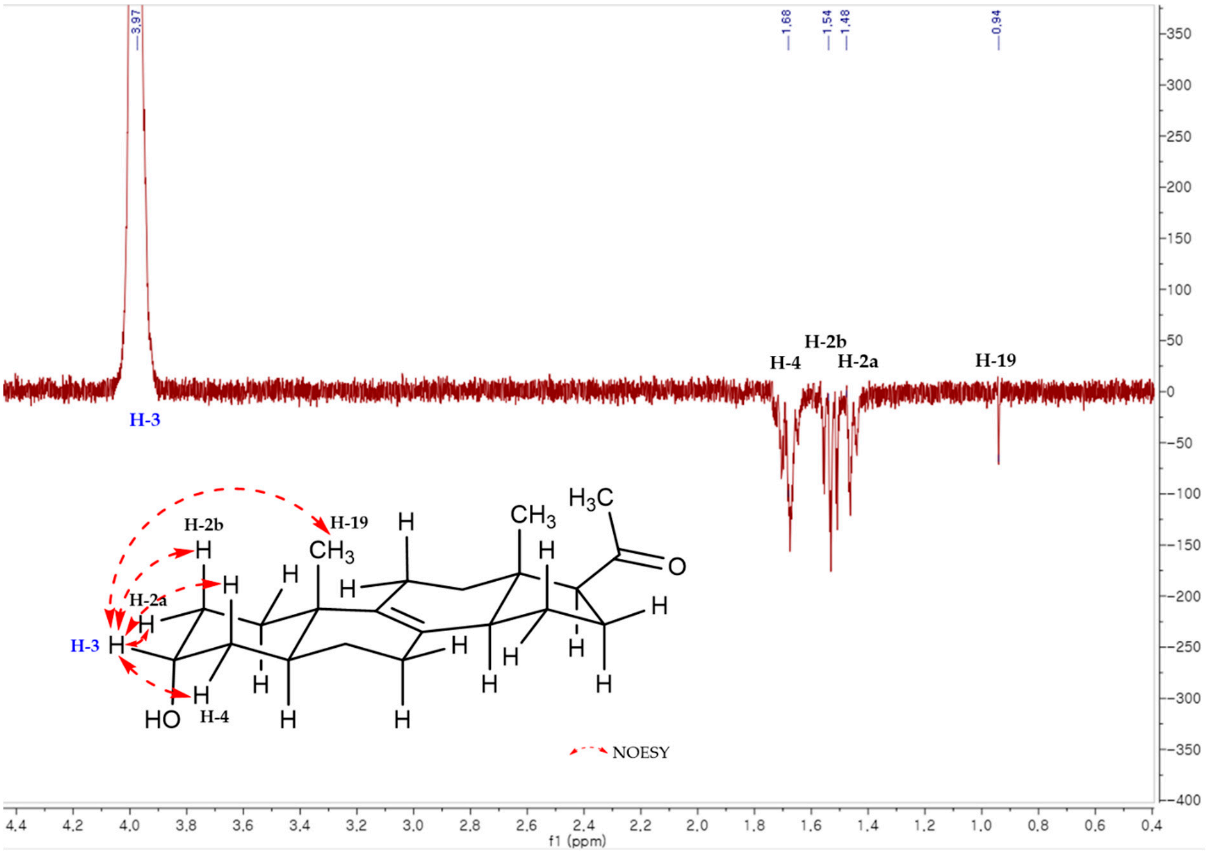


Figure S27. Selective 1D NOESY spectrum of **4** in $CD_3OD$.

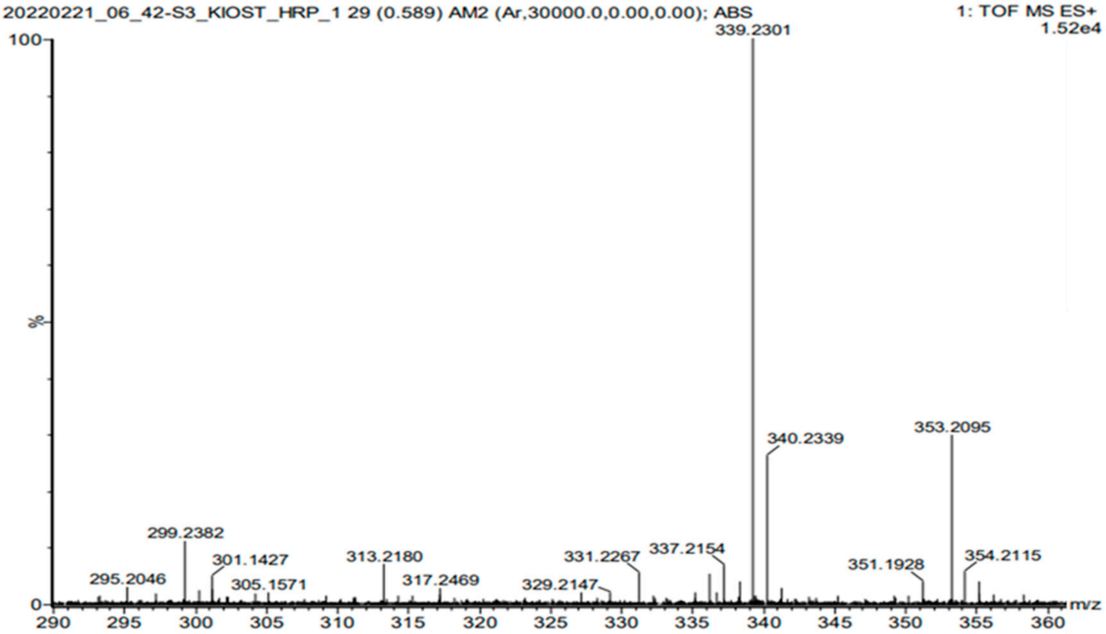


**Elemental Composition Report**

Single Mass Analysis
Tolerance = 5.0 PPM / DBE: min = -3.0, max = 200.0
Element prediction: Off
Number of isotope peaks used for i-FIT = 3

Monoisotopic Mass, Even Electron Ions
68 formula(e) evaluated with 1 results within limits (all results (up to 1000) for each mass)
Elements Used:
C: 0-31 H: 0-50 O: 0-7 Na: 0-1

| | | | | | | | | |
|---|---|---|---|---|---|---|---|---|
| Minimum: | | | | -3.0 | | | | |
| Maximum: | | 300.0 | 5.0 | 200.0 | | | | |
| Mass | Calc. Mass | mDa | PPM | DBE | i-FIT | Norm | Conf(%) | Formula |
| 339.2301 | 339.2300 | 0.1 | 0.3 | 5.5 | 562.8 | n/a | n/a | C21 H32 O2 Na |

Figure S28. HR-ESIMS spectrum of **4**.

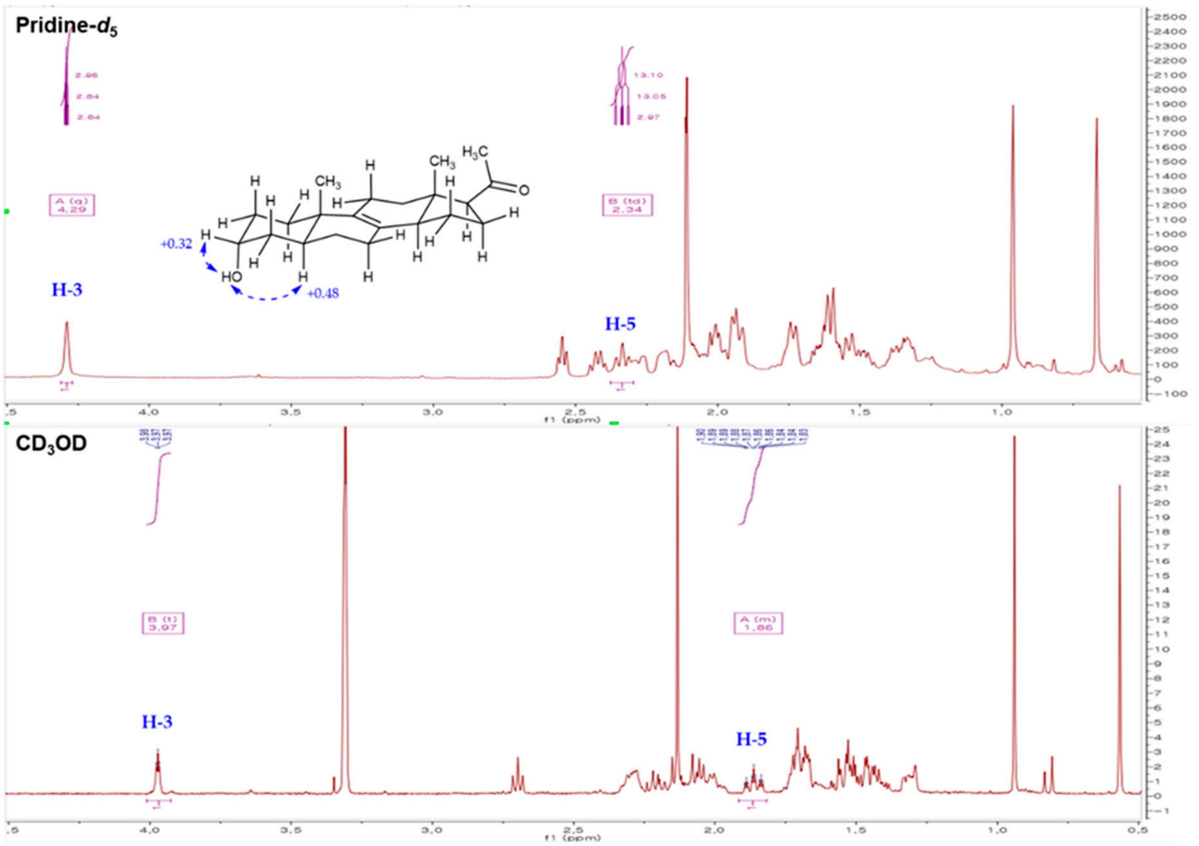


Figure S29. Comparison of $^1H$ NMR spectra of **4** in pyridine-$d_5$ and in $CD_3OD$.

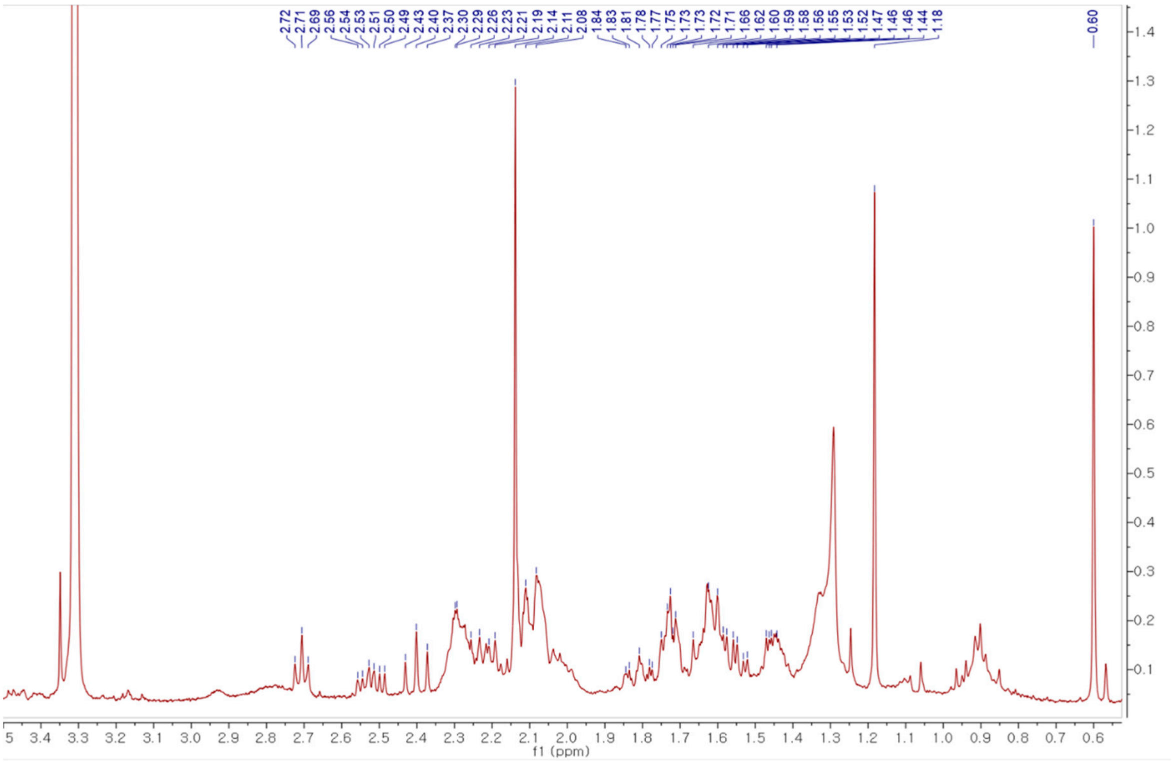


Figure S30. $^{1}H$ NMR spectrum of **5** in $CD_3OD$ (600 MHz)

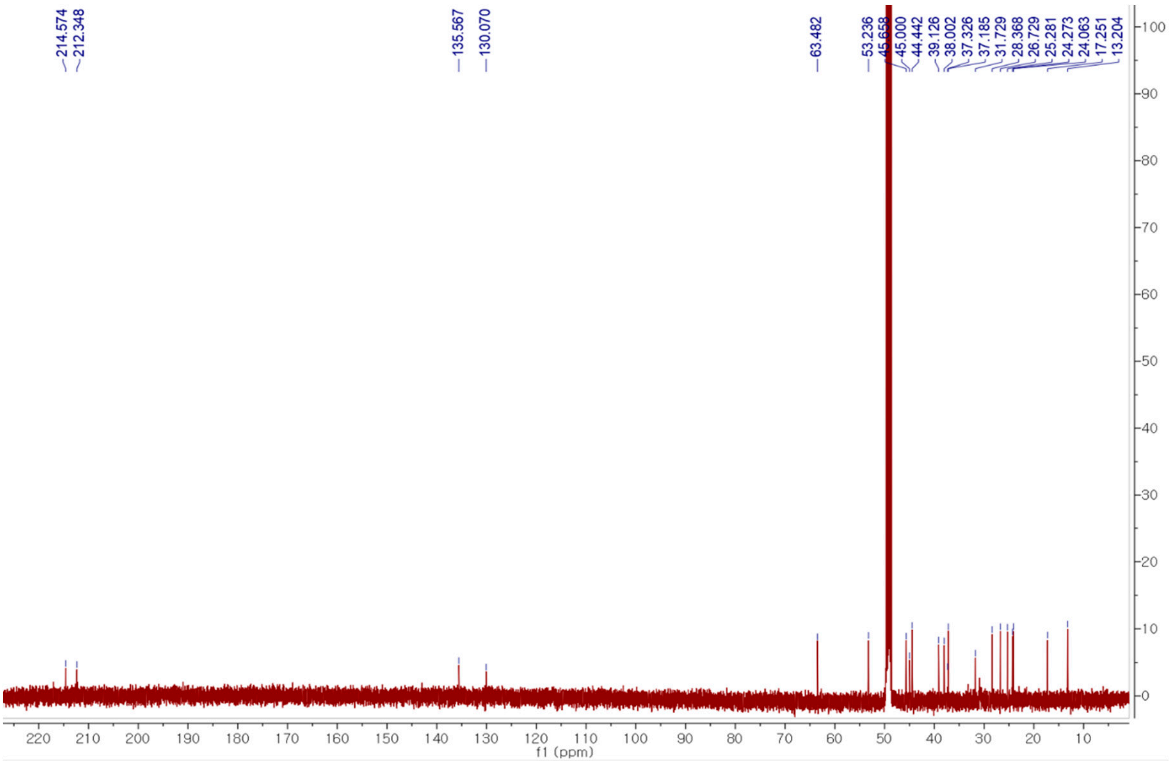


Figure S31. $^{13}C$ NMR spectrum of **5** in $CD_3OD$ (150 MHz).

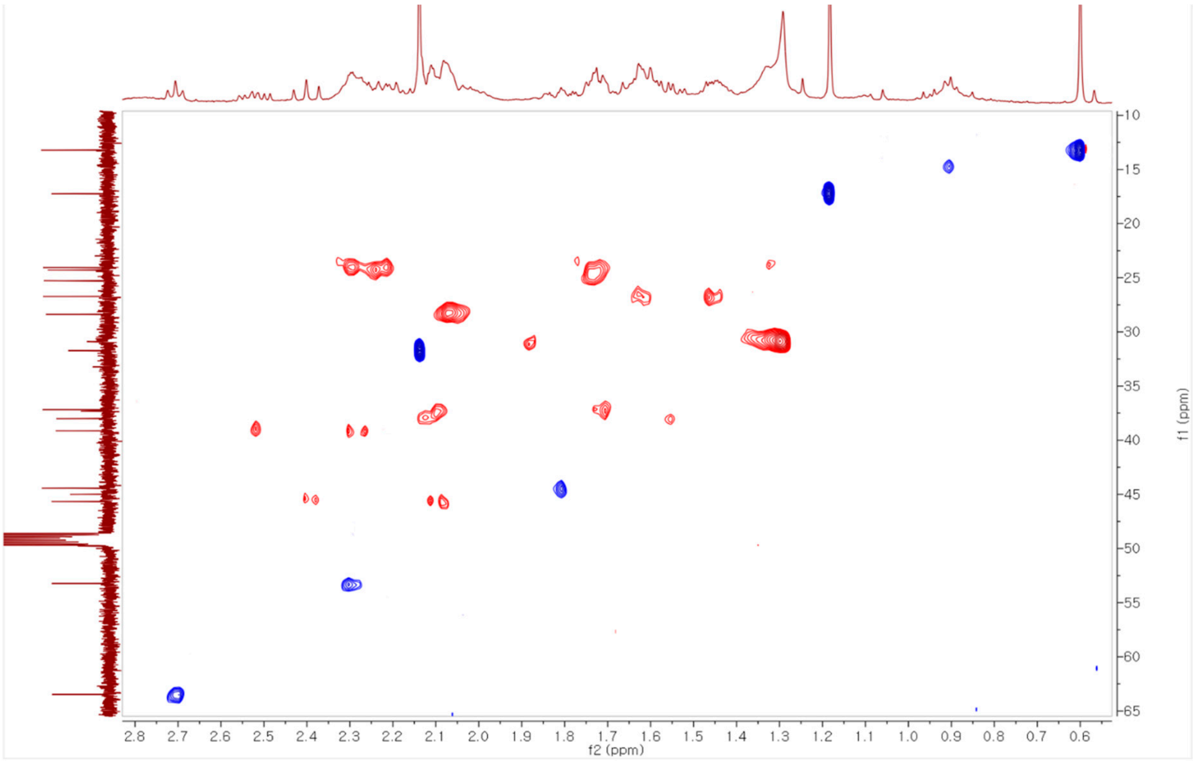


Figure S32. HSQC spectrum of **5** in $CD_3OD$.

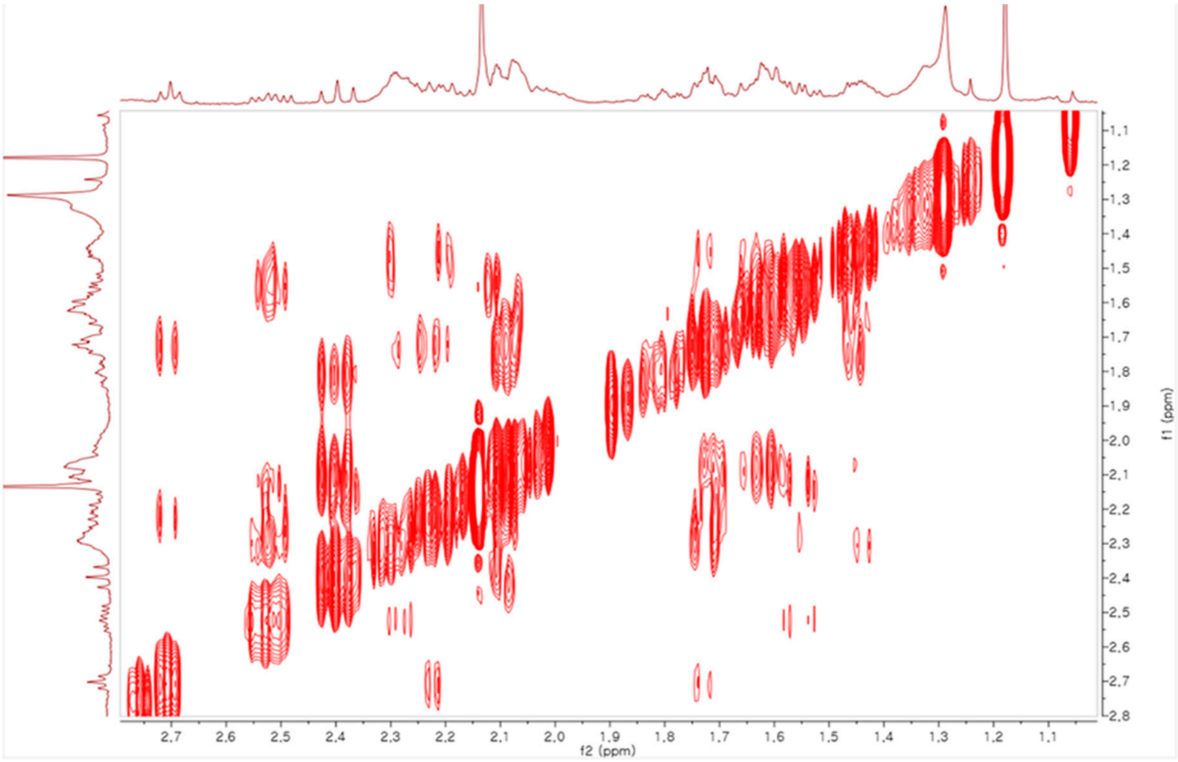


Figure S33. COSY spectrum of **5** in $CD_3OD$.

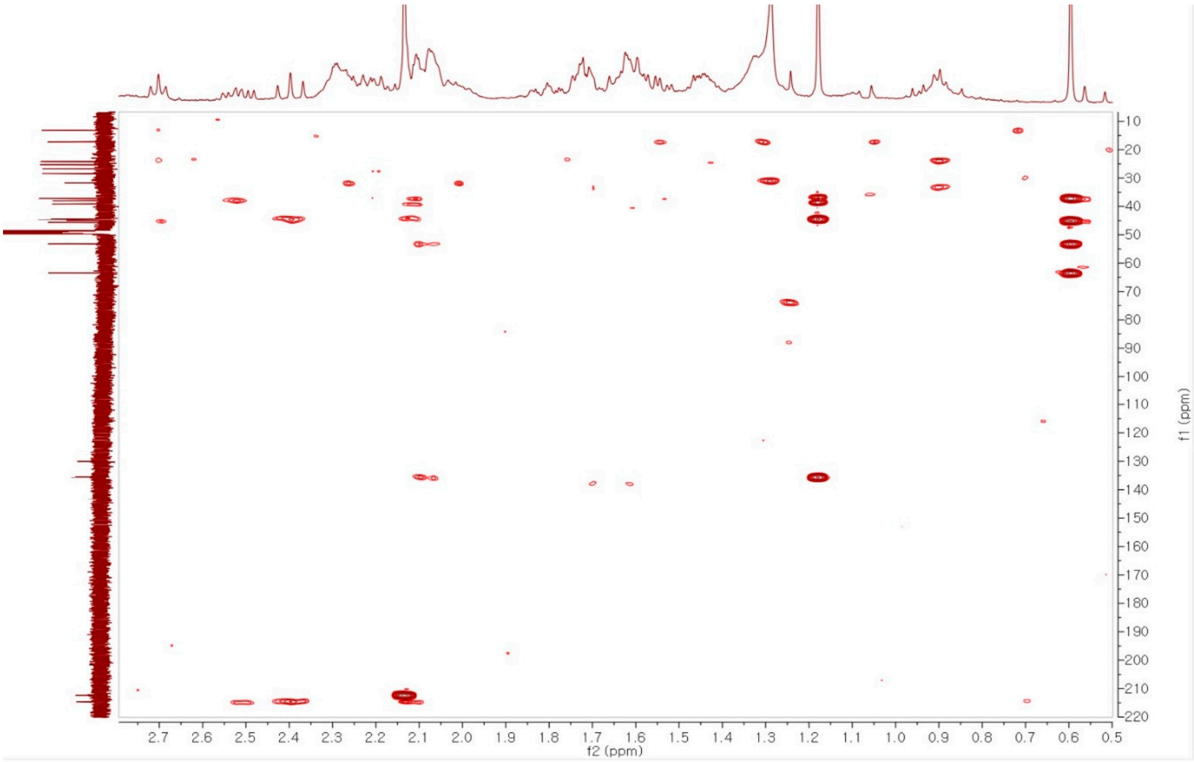


Figure S34. HMBC spectrum of **5** in $CD_3OD$.

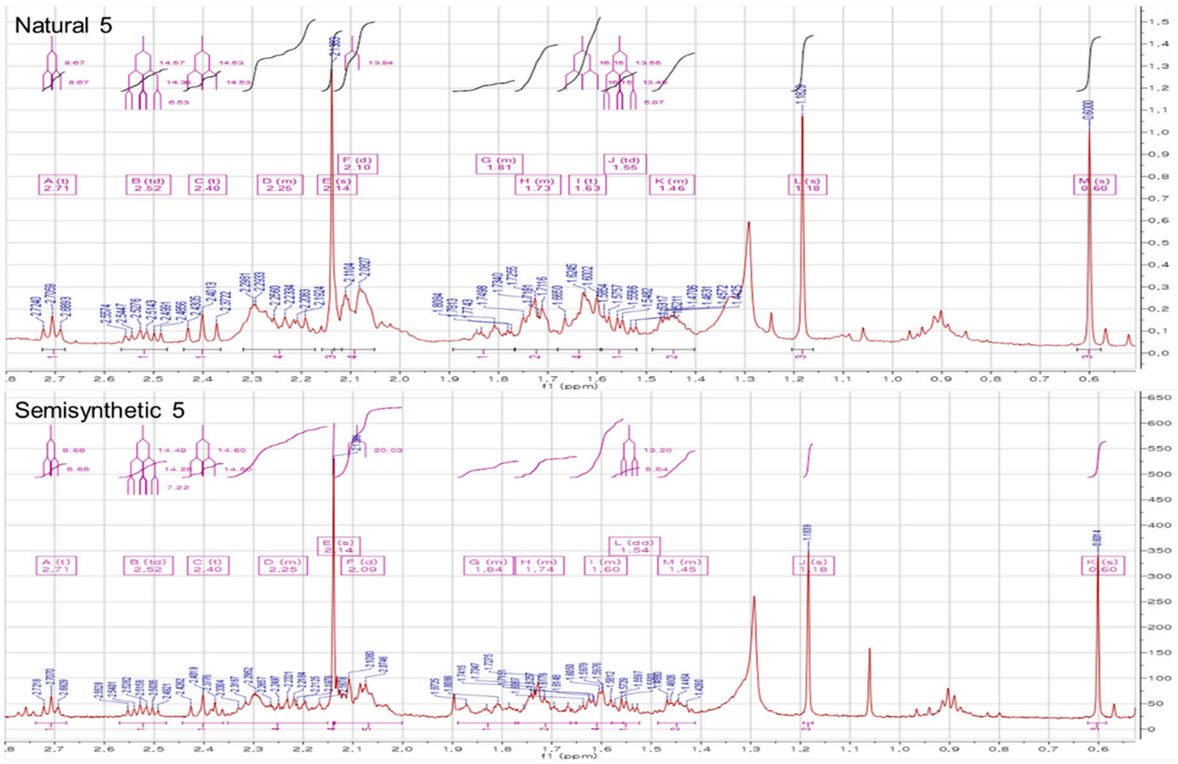


Figure S35. Comparison of $^{1}$H NMR spectra between natural **5** and semisynthetic **5** in $CD_3OD$ (600 MHz).

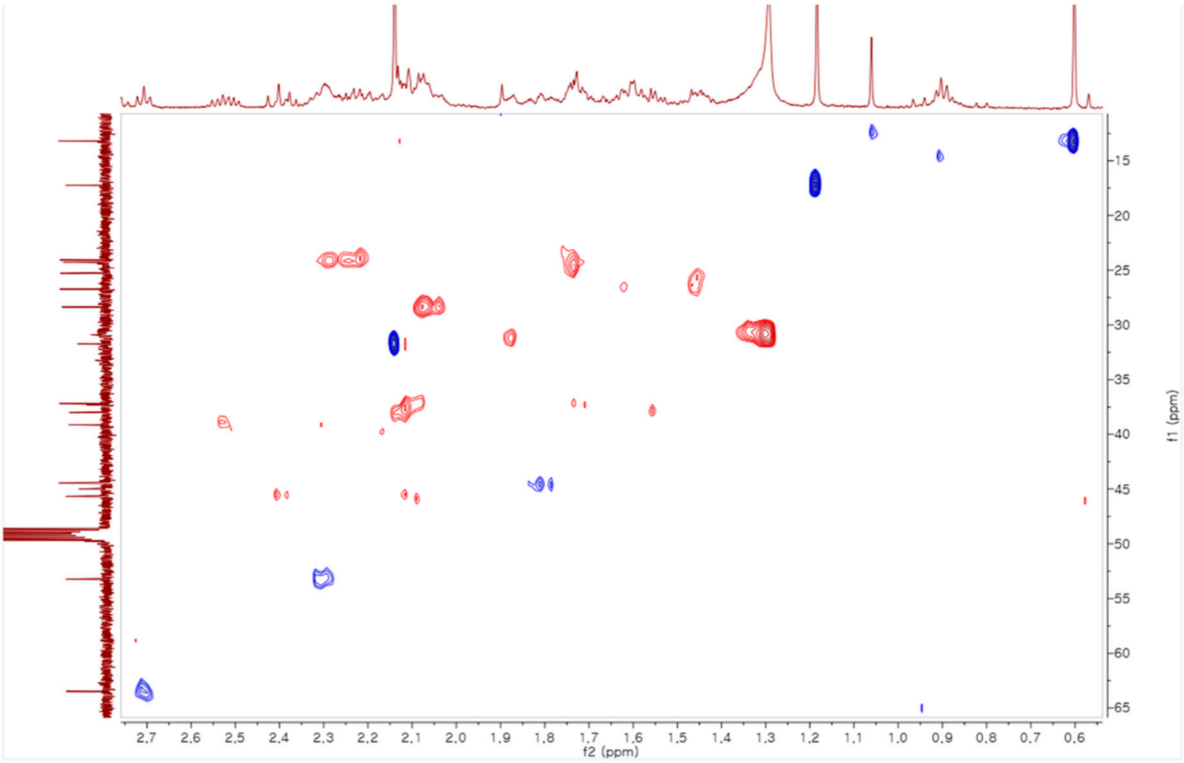


Figure S36. HSQC spectrum of semisynthetic **5** in $CD_3OD$.

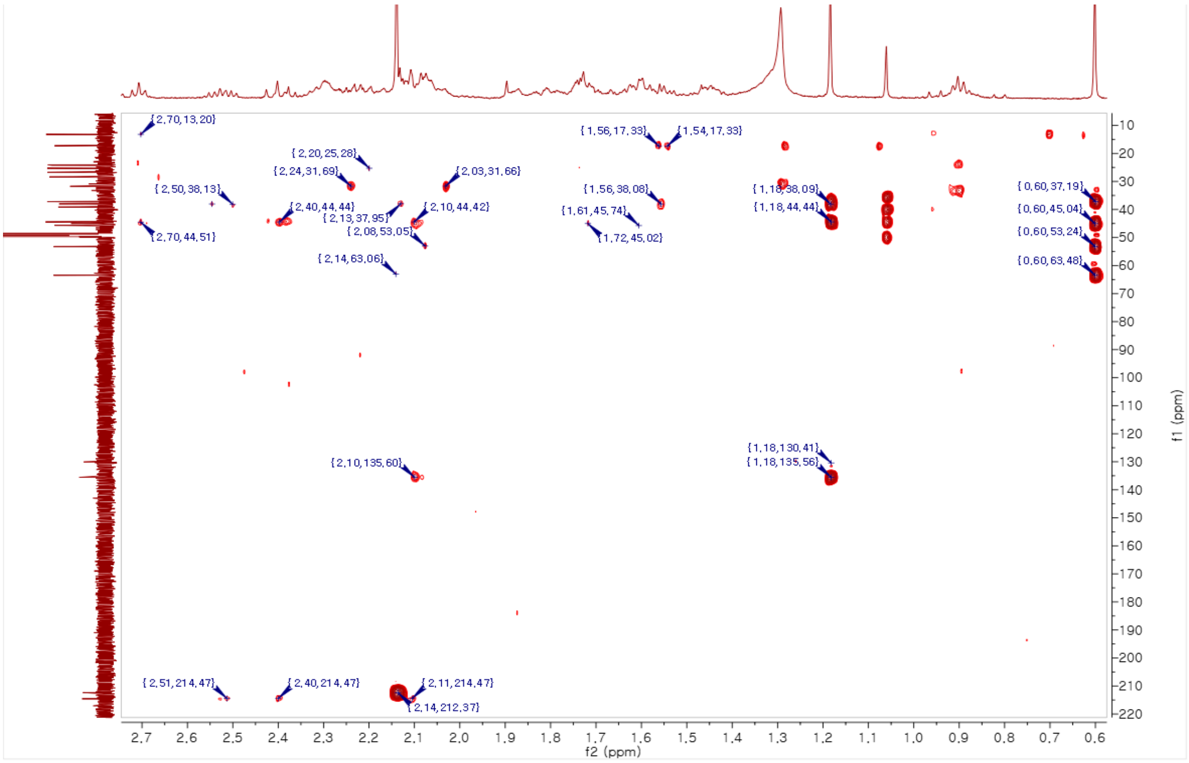


Figure S37. HMBC spectrum of semisynthetic **5** in $CD_3OD$.

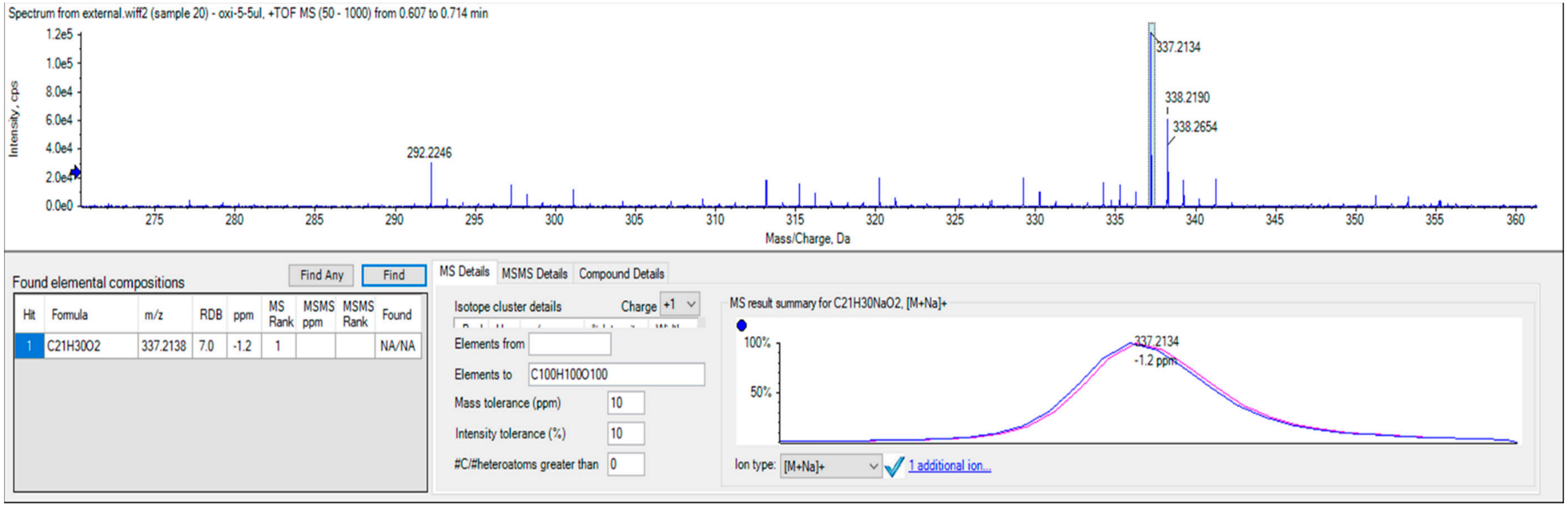


Figure S38. HR-ESIMS spectrum of semisynthetic **5.**
